# Use of subject-specific models to detect fatigue-related changes in running biomechanics: a random forest approach

**DOI:** 10.3389/fspor.2023.1283316

**Published:** 2023-12-21

**Authors:** Hannah L. Dimmick, Cody R. van Rassel, Martin J. MacInnis, Reed Ferber

**Affiliations:** ^1^ Human Performance Laboratory, Faculty of Kinesiology, University of Calgary, Calgary, AB, Canada; ^2^ Running Injury Clinic, Calgary, AB, Canada

**Keywords:** running biomechanics, machine learning, subject-specific model, running fatigue, wearable technology

## Abstract

Running biomechanics are affected by fatiguing or prolonged runs. However, no evidence to date has conclusively linked this effect to running-related injury (RRI) development or performance implications. Previous investigations using subject-specific models in running have demonstrated higher accuracy than group-based models, however, this has been infrequently applied to fatigue. In this study, two experiments were conducted to determine whether subject-specific models outperformed group-based models to classify running biomechanics during non-fatigued and fatigued conditions. In the first experiment, 16 participants performed four treadmill runs at or around the maximal lactate steady state. In the second experiment, nine participants performed five prolonged runs using commercial wearable devices. For each experiment, two segments were extracted from each trial from early and late in the run. For each participant, a random forest model was applied with a leave-one-run-out cross-validation to classify between the early (non-fatigued) and late (fatigued) segments. Additionally, group-based classifiers with a leave-one-subject-out cross validation were constructed. For experiment 1, mean classification accuracies for the single-subject and group-based classifiers were 68.2 ± 8.2% and 57.0 ± 8.9%, respectively. For experiment 2, mean classification accuracies for the single-subject and group-based classifiers were 68.9 ± 17.1% and 61.5 ± 11.7%, respectively. Variable importance rankings were consistent within participants, but these rankings differed from each participant to those of the group. Although the classification accuracies were relatively low, these findings highlight the advantage of subject-specific classifiers to detect changes in running biomechanics with fatigue and indicate the potential of using big data and wearable technology approaches in future research to determine possible connections between biomechanics and RRI.

## 1. Introduction

Researchers have long hypothesized that fatigue during running could be a contributing factor to running-related injury (RRI) (1, 2) and performance degradation (3–6). Fatigue-related changes in biomechanics may lead to tissues (i.e., bones, tendons, ligaments, muscles) receiving “atypical” stress/impact beyond the tissue's tensile limits (7). Given that “typical” biomechanics do not appear to be associated with injury (8) or performance (9, 10), it has been postulated that altered biomechanics due to fatigue could be “improper” for the body, leading to negative outcomes. Thus, if the nature of these atypical movements could be identified, runners could attempt to correct these alterations to maintain non-fatigued biomechanics and avoid injurious/inefficient movement patterns through strength and conditioning interventions. The ability to identify when biomechanics begin to change could also inform training and recovery protocols. However, to date, the scientific literature has not substantiated a consistent relationship between biomechanical patterns and RRI reduction or performance enhancement (11, 12), necessitating new approaches to understand the etiology and implications of fatigue on running biomechanics.

Conflicting results between physiological fatigue and concomitant changes in running biomechanics could be explained by methodological issues. One possibility is the heterogeneous nature of the studies (i.e., varying fatiguing protocols, participant populations, and data collection modalities), making synthesis difficult. Another potential factor is the predominance of group-based analyses (13, 14) that often ignore relevant individual responses. For example, previous studies have reported that fatigue is associated with significant increases in mean step length (15) and instantaneous loading rate (16), while simultaneously reporting that some individuals demonstrated opposite changes or no changes in those variables. Others have reported no changes in peak positive tibial acceleration, even though ∼50% of the participants showed a positive change, while others showed a negative change (17), washing out any reportable mean effect. These examples and others make it clear that group-based analyses can mask relevant responses from individual runners, obscuring meaningful conclusions.

In recent years, the medical field has turned towards “precision medicine”—a model based on “an understanding of the genetic make-up, personal lifestyle, gene, and surrounding environment of an individual” (18). This model shifts the focus from the “average” patient to the individual patient (19). Although some researchers have commented on the need for individualized models in a sports science context (20–22), noting “the “average” runner (mean data) [does] not resemble any of the individuals in a group”, (23), the volume of data required to design these models has, until recently, been difficult to obtain. However, with the advent and expanding accessibility of high-frequency data from wearable sensors and machine learning models (24), “precision sports science” (25) is an increasingly plausible and potentially useful framework (26).

At present, machine learning models have been used infrequently to investigate subject-specific models of running biomechanical fatigue. Buckley et al. (27) used a random forest classifier to distinguish fatigued and non-fatigued running biomechanics from a single center-of-mass (CoM) inertial measurement unit (IMU) in both subject-specific and group-based classifiers. The subject-specific classifiers out-performed the group-based classifier with accuracies of 89% and 75%, respectively. In contrast, Op De Beéck et al. (28) determined that individual models had slightly higher mean average error than group-based models when regressing rate of perceived exertion (RPE) to biomechanical features over the course of a fatiguing trial. These conflicting results may be due to analysis differences, with one using a binary fatigue classifier and the other employing a regression approach. Subject-specific models have significantly outperformed group-based models for differentiating incline (29) and weather (30) conditions during running. Furthermore, Ahamed et al. (30) demonstrated that in a random forest model, the most important features to classification were different between participants, indicating that biomechanics changed between conditions, but that these specific changes were largely unique to each individual. Determining feature importance for individuals during fatigue in running has not, to our knowledge, been investigated previously. Moreover, determining whether individuals rely on different strategies when adjusting biomechanics during fatigue in running is critical to ultimately determining the necessity of subject-specific intervention.

Therefore, the purpose of this study was to investigate the difference between subject-specific and group-based models used to classify the effect of exercise-induced fatigue from prolonged running in two experimental conditions: a lab-based treadmill protocol and an outdoor, “real-world” dataset. Describing biomechanical changes due to running fatigue at an individual level has potential implications for RRI prevention and performance if these effects can be correctly and consistently defined. We hypothesize that subject-specific models will show greater accuracy than group-based models and that data collected from the treadmill in controlled conditions will produce a model with higher accuracy than data collected from runners in real-world, outdoor conditions.

## 2. Methods

Two experiments were conducted for this investigation. Experiment 1 was performed using a laboratory-based protocol. Subsequently, Experiment 2 was developed to compare/complement the results from Experiment 1 using “in the wild” data.

### 2.1. Experiment 1

#### 2.1.1. Participants

Sixteen recreationally and competitively trained runners (7 female, 9 male, age = 30 ± 4 years, height = 174.3 ± 9.1 cm, weight = 70.5 ± 10.5 kg) provided informed consent to participate in this study, which was approved by the Ethics Board at the University of Calgary (REB20-0111). Participants were included if they were between the ages of 18 and 45 years and had a recent 10-km performance of ≤50 min or ≤55 min for men and women, respectively. All participants were familiar with treadmill running, were free of medical conditions and injuries that could interfere with metabolic and cardiorespiratory exercise responses and completed the Physical Activity Readiness Questionnaire prior to exercise to ensure there were no identifiable contraindications to exercise.

#### 2.1.2. Protocol

Participants visited the lab 5 times. For all visits, participants were instructed to refrain from eating and consuming caffeine at least 2 h prior to testing and to refrain from strenuous exercise at least 6 h prior. Participants used their own running shoes but were required to use the same shoes for each visit. In the first visit, maximal lactate steady state (MLSS) was estimated using a modified Step-Ramp-Step test that has been validated for use in running (31, 32). The MLSS represents the boundary between heavy and severe exercise (33–36). For the next 4 visits, participants performed treadmill runs at or around the estimated MLSS (2 trials at MLSS, 1 trial 5% above MLSS, 1 trial 5% below MLSS) (33). Each trial began with the participant performing a 5-min warmup at 1.9 m/s before increasing to the target speed. The trial was terminated when the participant reached volitional exhaustion, or at 45 min, whichever occurred first. Each visit was separated by at least 48 h, and participants were not informed of the speed until after all experimental trials were completed. During each trial, participants were fitted with an IMU (Blue Trident, Vicon, Oxford, UK; tri-axial accelerometer sampling rate 1,125 Hz, range ±16 g) positioned between the posterior superior iliac spines with the top border of the sensor positioned on a line coincident with the inferior aspect of the iliac crest. The X, Y, and Z axes were oriented in the vertical (+ to the superior), medial-lateral (+ to the left), and anterior-posterior (+ to the posterior) directions, respectively. Incline was set at 1% to correspond to previous literature (37). RPE values (38) were recorded every 5 min.

#### 2.1.3. Data processing

Initial contact for each step was identified using methods described in Benson et al. (39). A step was defined as the duration between consecutive initial contacts from contralateral feet, and a stride was defined as the duration between consecutive initial contacts from the ipsilateral foot. Mean and standard deviation of the number of data points in each step were calculated, and those ±2 standard deviations from the mean were labeled as improperly segmented and excluded (39). Samples were constructed from the raw signal of five consecutive strides (39, 40). Non-fatigue (NF) and fatigue (FT) conditions were considered the first 5 min of the trial and the final complete 5-min segment (to correspond to RPE sampling), respectively (41). The RPE label for each condition was the value provided at the end of the 5-min segment. For example, if a participant terminated the test at 38 min, the period from 30 to 35 min would be defined as the FT condition, and it would correspond to a single RPE value (taken at 35 min).

Thirty-nine features were extracted from the acceleration signals of each sample: mean, standard deviation, median, 25th percentile, 75th percentile, root mean square (RMS), maximum, minimum, sample entropy calculated from the three primary axes (vertical, medio-lateral, anterior-posterior) and the resultant (17, 28, 39, 42–48), and the ratio of single-axis RMS to resultant RMS (49). These features were selected based on previous analyses (31) and were identified due to their typical inclusion as features in machine learning models.

### 2.2. Experiment 2

#### 2.2.1. Participants

Nine participants (1 female, 8 males, age = 44 ± 13 years) were selected from the Wearable Technology Citizen Science Level-4 secure research database on the following criteria: reported very good or excellent health in the previous year, recorded at least 5 outdoor runs that were >14 km within a 3-month period, and recorded runs with a qualifying Garmin device (Garmin, Inc., Olathe, KS, USA). Participants provided informed consent to share their information through the database, which was approved by the Ethics Board at the University of Calgary (REB20-0572).

#### 2.2.2. Protocol

Within the database, the frequency of activities, weather, distance, route, speed, and surfaces represent individuals' own training habits and were not prescribed or controlled by researchers. Runs selected for this study were required to be completed outdoors, within a 3-month period, under similar weather conditions to minimize variability (e.g., effects of training) (30). Surfaces were not reported, and injury history/training status were not known.

Participants used their own Garmin devices for data collection (HRM-Tri, *n* = 5; HRM-Run, *n* = 3; Running Dynamics Pod, *n* = 1). Qualifying devices were those that were (1) enabled to calculate Garmin Running Dynamics and (2) had been previously lab-validated (50, 51). The HRM devices are mounted on the sternum and the Running Dynamics Pod is mounted on the low back. Garmin Running Dynamics are variables calculated onboard select Garmin devices: stance time, stance time balance, cadence, stride length, vertical oscillation, and vertical ratio (52). Each participant used the same device for every run that was included in analysis. Data were sampled using the device's onboard algorithms, uploaded by the participant to the Garmin Connect online platform, and extracted for data processing via the Wearable Technology Citizen database.

#### 2.2.3. Data processing

Five of 6 Garmin Running Dynamics variables were selected for analysis: stance time, stance time balance, cadence, stride length, and vertical oscillation. It should be noted that vertical ratio was excluded due to its direct correlation to vertical oscillation and stride length (i.e., it would add minimal additional relevant information for classification). Trials were only included if <20% of strides had any variable missing. Strides with any missing variables were excluded from analysis. Trials were additionally excluded if the analysis segments included stopped time (e.g., for a walk break or red light) ≥ 30 s. This selection process was performed until each participant had 5 runs that met the criteria.

To remove the effect of speed and grade, which can have significant impacts on biomechanics (53, 54), all variables were analyzed in successive univariate models adjusted for speed and grade (55).

**Figure 1:**
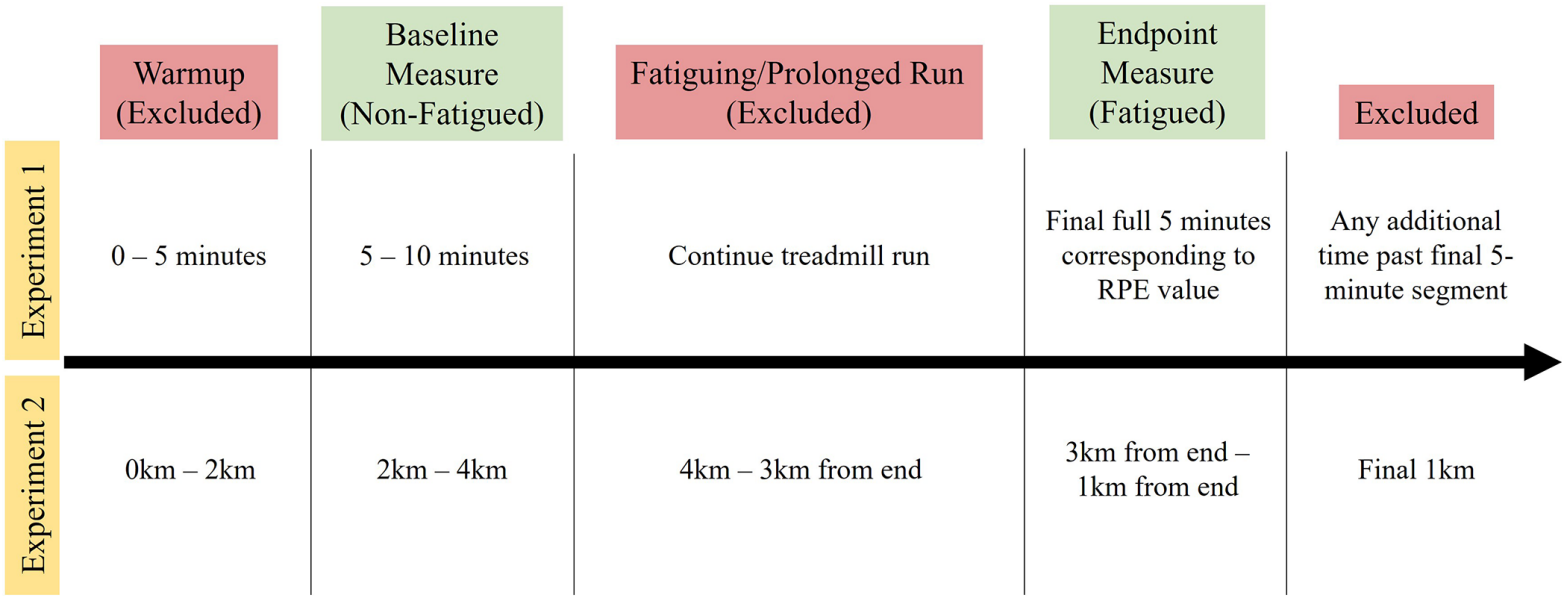
Timeline of segment selection.

The first 2-km and final 1-km were excluded from each trial to account for any potential changes in biomechanics associated with warmup or cooldown. After these exclusions, the first 2-km segment (BEG) and the final 2-km segment of each run were selected for analysis. A visualization of the segment selection from both experiments can be found in Figure 1. Due to the constraints of the database, participants' fatigue level in Experiment 2 could not be empirically determined. The minimum distance, 14-km, was selected to ensure reasonable confidence that participants would be experiencing fatigue by the end of their session. For simplicity, this prolonged effort is referred to here as “fatiguing”.

### 2.3. Data analysis

An ensemble machine learning classifier, random forest, was employed to develop classification models for the NF vs. FT during Experiment 1, as well as the BEG vs. END segments for Experiment 2. The random forest classifier provides high levels of accuracy while additionally reporting the relative importance of each predictor variable (56). The random forest model was selected for several reasons. First, in a pilot analysis, random forest performed as well or better than a support vector machine, naïve bayes, and logistic regression. Additionally, the random forest allowed for consistency with previous literature (29, 30) and robustness to small datasets (56).

**Figure 2:**
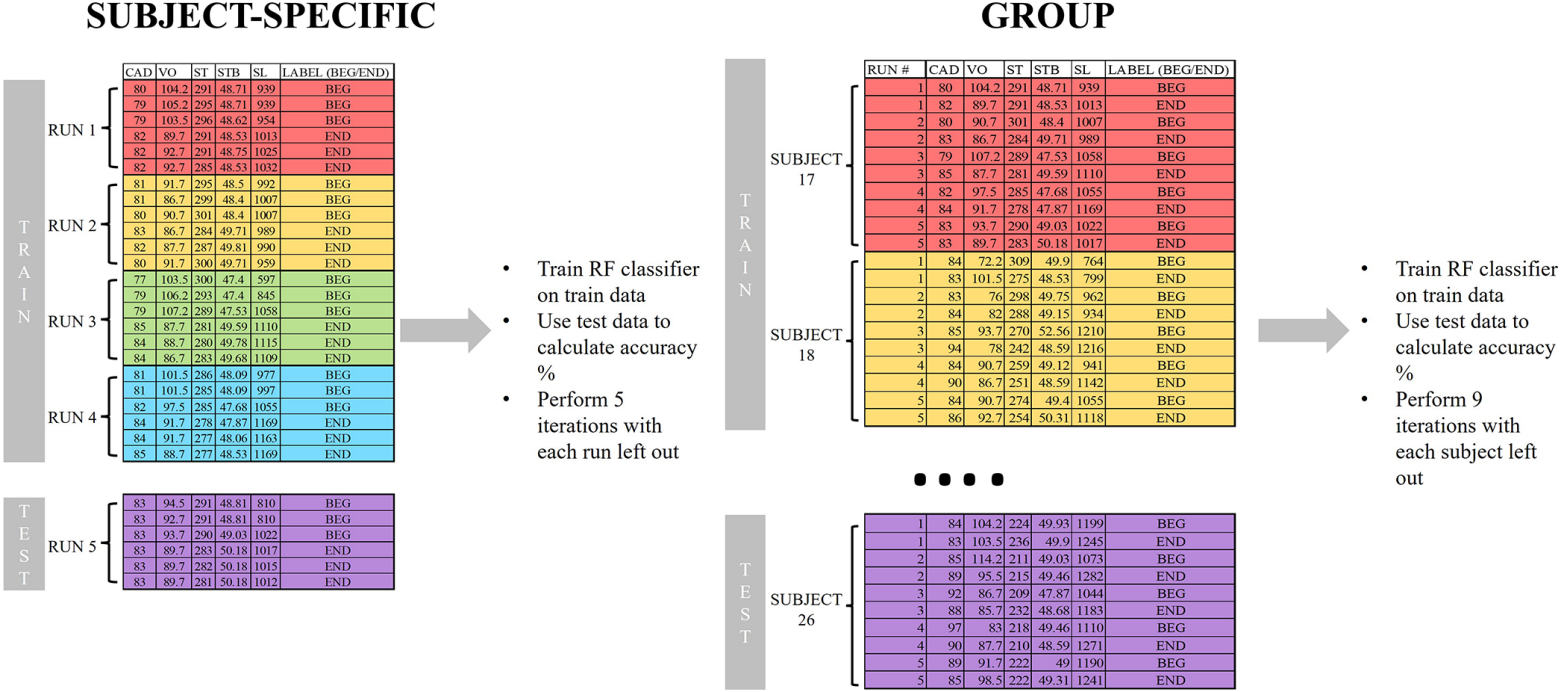
Example data from Experiment 1 to demonstrate the workflow of the random forest classifiers for subject-specific and group-based models.

Two methods were used to determine the ability of the random forest classifier to distinguish between the two conditions (57, 58). These methods are described in Figure 2. Method 1 was a subject-specific approach, where data from only one participant at a time was included. A leave-one-trial-out cross-validation was performed. Data from 3 (Experiment 1) or 4 (Experiment 2) runs were used as the training set, and the test set consisted of the final (4th/5th) run. This was performed 4–5 times per participant, with each possible combination of runs used for the training and test sets.

Method 2 was a group-based approach and collapsed all runs for each participant into a single set per participant. Then, a leave-one-subject-out cross-validation was performed by using data from *n-1* participants were used as the training set, with the test set consisting of the left-out participant. This was performed for each participant. Both methods were performed for each segment pair (NF vs. FT, BEG vs. END) and were applied using the standalone Python programming language (version 3.7.4, www.python.org) (59).

The developed random forest models were tuned with a grid search (*max_depth* (2–20), *n_estimators* (100–1000), and *max_features* (1–39 for Experiment 1, 1–5 for Experiment 2)) on the training set using a 5-fold cross-validation with the built-in Anaconda distribution (open-source) of Python programming language, using numpy, scikit-learn, and scipy (“sklearn.ensemble.RandomForestClassifier”) packages (60). Because all variables were continuous, the random forest employed a Gini index calculation to determine variable importance based on impurity reduction (61).

For further information on usage of random forest for commercial wearable data, refer to Ahamed et al. (30).

Significance of classification results was tested using binomial methods from Combrisson & Jerbi (62)*.* These methods provide a framework to perform empirical significance testing for binary classifiers given limitations in sample size, rather than assuming a theoretical significance of >50% accuracy.

To compare RPE between conditions in Experiment 1, a paired samples *t*-test was performed.

Significance was set at *α* ≤ 0.05.

## 3. Results

### 3.1. Descriptive

Trial details for each participant are presented in Tables 1, 2 for Experiments 1 and 2, respectively. For Experiment 1, treadmill speeds at MLSS ranged from 2.77–3.98 m/s (males = 2.77–3.98 m/s, females = 2.82–3.89 m/s). Results of other tested classifiers are reported in Supplementary Tables S1, S2.

**Table 1:** Descriptive characteristics of participants and included runs for Experiment 1; mean ± SD (range).

Participant	Sex	Age (years)	Trial time (min)	Mean strides per run (NF)	Mean strides per run (FT)	RPE_NF_	RPE_FT_
1	M	28	44.7 ± 0.6 (43.8–45.0)	407.5 ± 13.2 (395–425)	390.0 ± 50.2 (315–420)	11.5 ± 0.6 (11–12)	12.8 ± 1.5 (11–14)
2	F	32	35.0 ± 7.1 (30.0–45.0)	370.0 ± 21.6 (340–390)	375.0 ± 47.4 (315–420)	12.8 ± 0.5 (12–13)	16.5 ± 1.3 (15–18)
3	M	24	38.8 ± 7.5 (30.0–45.0)	377.5 ± 53.6 (330–450)	422.5 ± 70.0 (320–475)	12.8 ± 2.1 (10–15)	17.8 ± 1.9 (15–19)
4	M	26	27.5 ± 5.0 (20.0–30.0)	373.8 ± 53.0 (315–430)	377.5 ± 68.4 (275–415)	11.8 ± 1.7 (10–14)	13.5 ± 1.3 (12–15)
5	M	25	32.9 ± 5.9 (26.6–40.0)	355.0 ± 67.6 (260–420)	278.8 ± 95.1 (190–390)	13.3 ± 1.0 (12–14)	17.5 ± 0.6 (17–18)
6	M	32	45.0 ± 0.0 (45.0–45.0)	377.5 ± 66.6 (325–475)	343.8 ± 56.5 (270–395)	10.3 ± 1.3 (9–12)	15.3 ± 1.7 (13–17)
7	M	29	37.5 ± 6.5 (30.0–45.0)	425.0 ± 123.4 (310–600)	350.0 ± 92.5 (270–395)	9.8 ± 0.5 (9–10)	14.3 ± 1.9 (13–17)
8	M	40	41.5 ± 4.4 (36.0–45.0)	410.0 ± 61.8 (350–495)	386.3 ± 67.5 (300–440)	12.3 ± 1.0 (11–13)	14.5 ± 1.3 (13–16)
9	F	25	43.3 ± 3.5 (38.0–45.0)	311.3 ± 200.1 (130–545)	368.8 ± 67.3 (275–420)	11.5 ± 1.3 (10–13)	15.3 ± 1.3 (14–17)
10	F	29	41.3 ± 7.5 (30.0–45.0)	291.3 ± 88.2 (160–350)	327.5 ± 57.8 (250–390)	11.8 ± 0.5 (11–12)	15.8 ± 1.0 (15–17)
11	F	34	35.6 ± 11.3 (22.5–45.0)	526.3 ± 207.6 (300–705)	372.5 ± 69.3 (310–435)	13.8 ± 0.5 (13–14)	16.0 ± 0.8 (15–17)
12	F	32	41.3 ± 7.5 (30.0–45.0)	367.5 ± 8.7 (355–375)	417.5 ± 8.7 (405–425)	9.0 ± 1.2 (8–10)	15.8 ± 2.2 (13–18)
13	M	33	38.9 ± 12.3 (20.5–45.0)	376.3 ± 55.1 (315–430)	383.8 ± 79.8 (305–455)	10.0 ± 0.8 (9–11)	14.5 ± 1.3 (13–16)
14	F	33	40.8 ± 8.5 (28.0–45.0)	508.8 ± 164.2 (420–755)	430.0 ± 13.5 (410–440)	13.0 ± 0.8 (12–14)	16.0 ± 1.4 (15–18)
15	F	27	33.9 ± 9.4 (23.2–45.0)	396.3 ± 30.7 (365–435)	403.8 ± 8.5 (395–415)	12.5 ± 0.6 (12–13)	16.5 ± 1.3 (15–18)
16	M	32	30.0 ± 0.0 (30.0–30.0)	423.8 ± 99.8 (290–530)	338.8 ± 30.7 (310–370)	9.8 ± 1.0 (9–11)	15.3 ± 1.3 (14–17)
Mean		30 ± 4.2	38.0 ± 7.9	393.6 ± 60.2	372.9 ± 39.0	11.6 ± 0.5	15.4 ± 1.8*

NF, non-fatigued segment; FT, fatigued segment; RPE, rating of perceived exertion.

* Significantly greater than RPE_NF_ (*p* < 0.05).

**Table 2:** Descriptive characteristics of participants and included runs for Experiment 2; mean ± SD (range).

Participant	Sex	Age (years)	Distance of runs analyzed (km)	Mean strides per run (BEG)	Mean strides per run (END)
17	M	43	25.5 ± 7.5 (16.1–32.5)	194.0 ± 18.9 (170–217)	222.0 ± 33.4 (184–273)
18	M	30	16.9 ± 3.3 (14.2–22.2)	675.2 ± 118.1 (535–820)	723.4 ± 87.8 (623–847)
19	F	43	18.2 ± 2.8 (15.8–22.1)	151.8 ± 17.0 (136–173)	139.6 ± 11.6 (130–158)
20	M	61	57.1 ± 8.4 (42.5–62.9)	355.6 ± 79.6 (263–474)	382.6 ± 260.2 (152–825)
21	M	38	22.4 ± 6.8 (15.7–30.0)	187.2 ± 15.6 (168–202)	207.8 ± 11.9 (191–219)
22	M	46	27.6 ± 11.0 (14.3––43.1)	134.2 ± 5.8 (130–144)	141.4 ± 12.9 (120–152)
23	M	61	14.8 ± 0.5 (14.0–15.3)	237.0 ± 17.7 (220–262)	235.8 ± 46.3 (167–289)
24	M	50	18.0 ± 4.2 (14.5–25.2)	123.2 ± 10.9 (105–130)	133.8 ± 33.2 (113–191)
25	M	24	16.7 ± 3.1 (14.6–22.0)	150.0 ± 12.1 (139–169)	152.0 ± 8.5 (144–166)
Mean		42 ± 12	24.1 ± 13.1	245.4 ± 176.1	259.8 ± 190.6*

BEG, beginning segment; END, end segment.

* Significantly greater than mean strides per run (BEG) (*p* < 0.05).

### 3.2. Random forest classifiers

**Figure 3:**
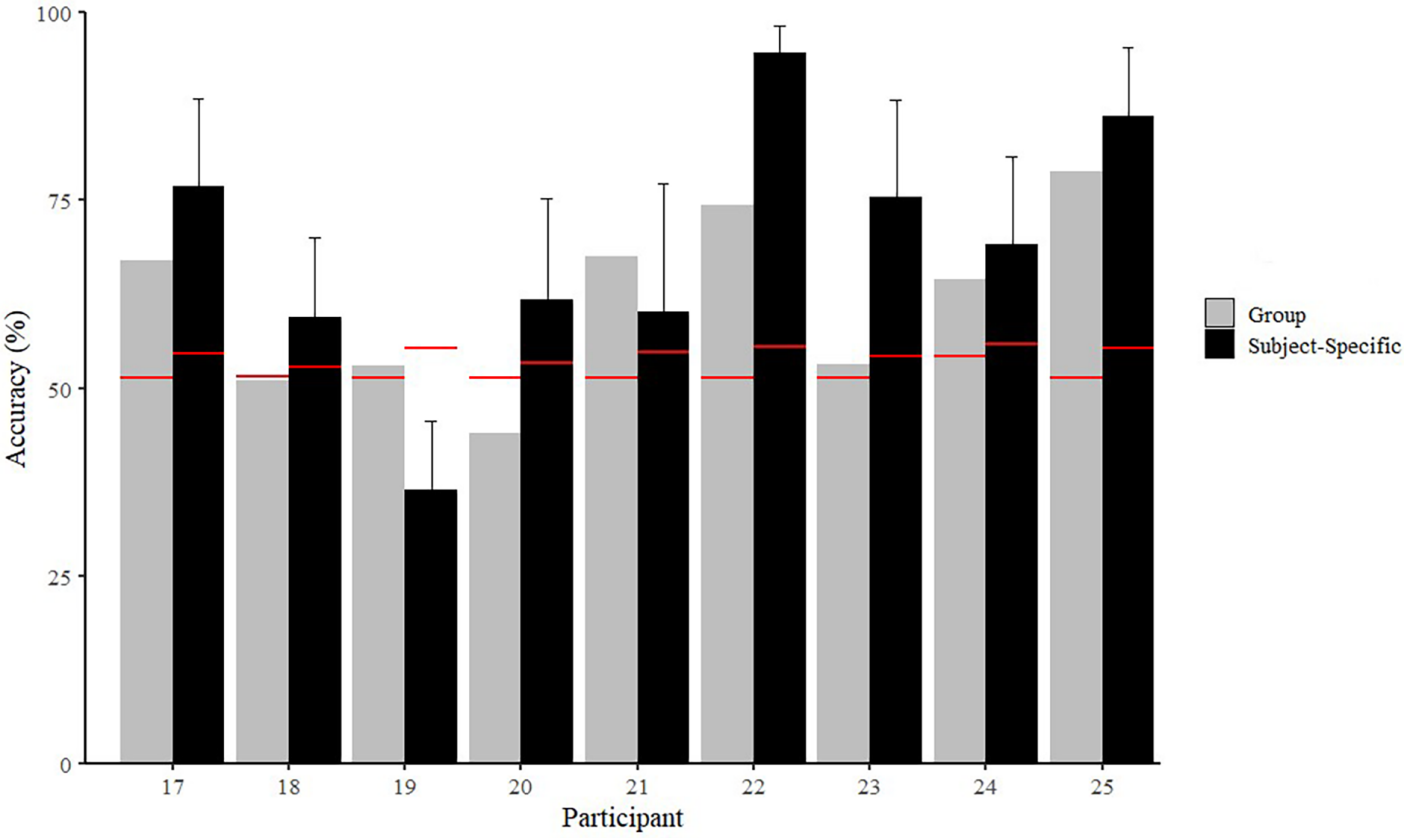
Accuracies of non-fatigued (NF) vs. fatigued (FT) classifiers for subject-specific and group-based models in Experiment 1. Group-based values reflect the accuracy of the model when the specific participant was used as the left-out test set. Red lines represent minimum classification accuracy required for significance (*p* < 0.05).

**Figure 4:**
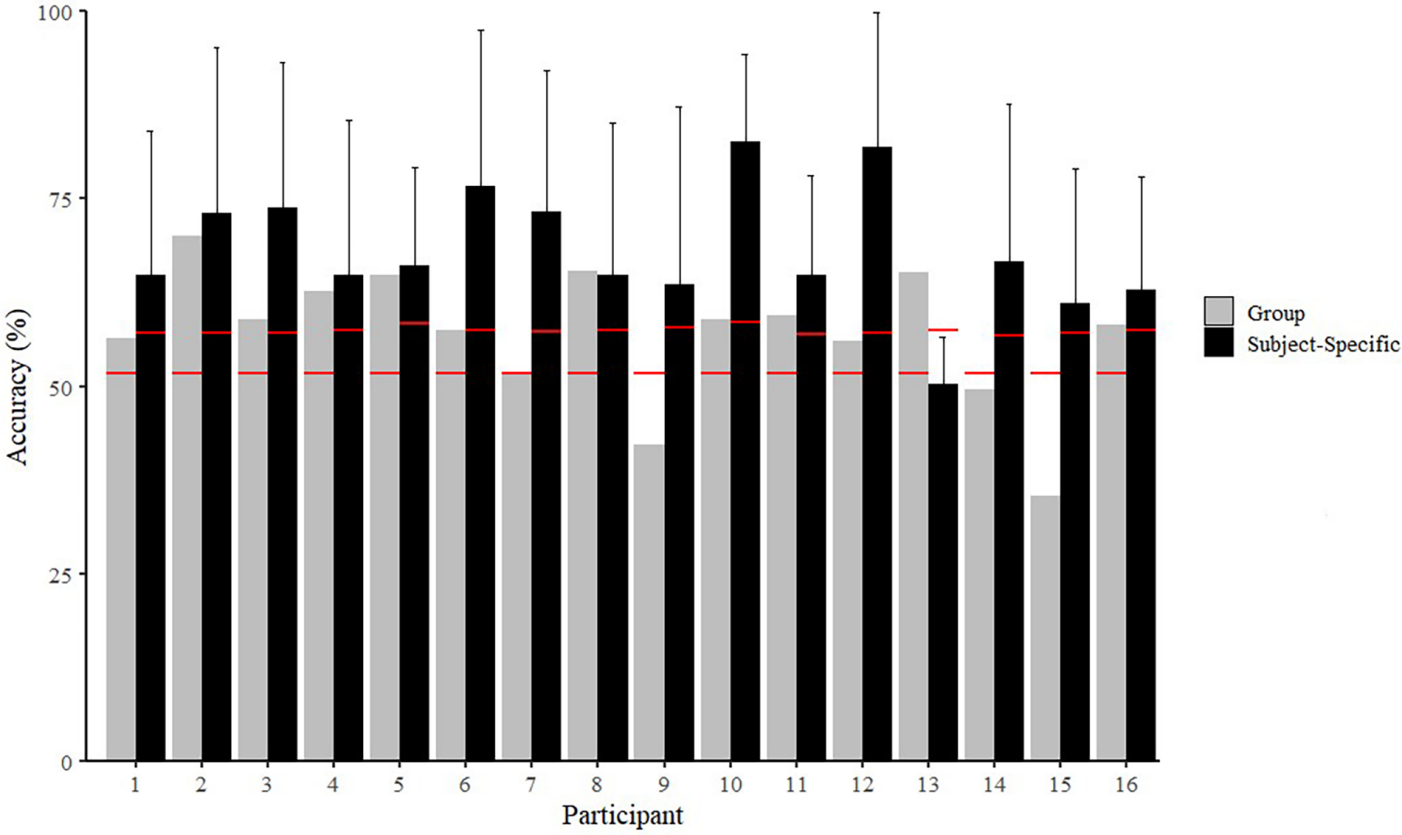
Accuracies of beginning (BEG) vs. end (END) classifiers for subject-specific and group-based models in Experiment 2. Group-based values reflect the accuracy of the model when the specific participant was used as the left-out test set. Red lines represent minimum classification accuracy required for significance (*p* < 0.05).

Figures 3, 4 present the results of the random forest classifiers for Experiments 1 and 2, respectively, and include the minimum classification accuracy required for significance. Mean classification accuracies for the single-subject and group-based NF vs. FT classifiers from Experiment 1 were 68.2 ± 8.2% and 57.0 ± 8.9%, respectively. Mean classification accuracies for the single-subject and group-based BEG vs. END classifiers from Experiment 2 were 68.9 ± 17.1% and 61.5 ± 11.7%, respectively. Further details of individual classifier results, including details on precision and recall, can be found in the Supplementary Tables S3–S6. Variable importance rankings are presented in Supplementary Tables S7–S10, and the general trend of the data indicated that individual participants showed relatively high consistency in variable importance rankings across classifier iterations (with different train/test sets), but these did not necessarily align with the variable importance rankings in the group-based model, or other individuals' subject-specific models. Alternatively, in the group-based models, variable importance rankings were generally similar no matter which participant was used as the test set.

For example, for every iteration of the BEG vs. END classifiers, the group-based model ranked cadence and stance time as the most important features, but Participant 23 ranked ST as the least important each time in the subject-specific models. Additionally, in the BEG vs. END subject-specific classifiers, Participant 6 never ranked stance time balance outside of the top 2, whereas Participant 25 only ranked stance time balance in the bottom 2. In the NF vs. FT classifier, the median of the vertical axis was selected in the top 5 for 14 out of 16 iterations in the group-based classifier, but was always ranked in the bottom 10 features for Participant 16.

## 4. Discussion

The primary finding of this study was that subject-specific models were more accurate than group-based models in distinguishing between non-fatigued and fatigued running states. These results support our hypothesis and align with previous findings that have demonstrated greater efficacy of subject-specific models over group-based classifiers for fatigue (27) and other running conditions (30, 63, 64). In contrast to our hypothesis, the mean classification accuracies were relatively similar between Experiments 1 and 2, with high variability between participants and trials. A secondary finding was that different variable importance rankings were observed between participants, as well as different rankings between each participant and the group classifiers.

The accuracy of the subject-specific classifiers here was lower than a previous study that also used individualized binary classifiers from an IMU at the CoM (89%) (27). However, that study used a different type of fatiguing protocol (beep test) and a continuous signal rather than a discrete or statistical feature set, so it may not be directly comparable to the current study. Moreover, the group-based accuracy means (i.e., NF vs. FT = 57.0 ± 8.9%; BEG vs. END = 61.5 ± 11.7%) and ranges (i.e., NF vs. FT = 35.3–70.1%; BEG vs. END = 44.0–78.8%) were largely similar to previous similar analyses using CoM-mounted IMUs to classify between non-fatigued and fatigued states (i.e., 60.9% (43); 53%–64% (27)).

Importantly, the classification accuracies of most models were statistically significant (*p* < 0.05), although this does not necessarily indicate that the accuracy was sufficient to be meaningful to an end user. As discussed previously, different types of runners may have different standards for accuracy, sensitivity, and specificity of a system that could be used to identify fatigue (43). Determining the minimum accuracy for usefulness may be a topic for future investigations. A system with high specificity but low sensitivity for identifying fatigue may not be sufficient for making real-time training decisions based on fatigue identification given the lack of ability to detect fatigue as it appears. However, a system with high sensitivity and low specificity may lead to disuse or overly cautious training decisions due to over-identification of fatigue states. There are several possible reasons that higher classification accuracy was not observed here. First, a single sensor placed at the CoM may be insufficient to detect global changes in biomechanics due to fatigue in these protocols. Marotta et al. (43) concluded that placing sensors on either side of a joint, especially the knee joint (i.e., thigh and shank), produced the highest classification accuracies between non-fatigued and fatigued biomechanics. Therefore, to adequately detect these effects, additional sensor information may be required. Secondly, previous studies have hypothesized that if the exercise intensity (and associated cardiovascular stress) is high enough, participants may terminate the activity prior to enough neuromuscular fatigue occurring to alter biomechanics (65). This could have been a factor in Experiment 1, especially for the trials at the highest speed, whereas in Experiment 2, because no information was collected on participants' fatigue states, it is possible that the prolonged runs were an insufficient fatiguing stimulus to adequately alter biomechanics as detected from a CoM IMU for some participants (2, 41, 66–69). More data/additional trials may also be required to adequately quantify an individual's fatigue response. Further research employing different sensor arrangements, number of running trials, and intensity of trials is therefore necessary.

Differences in fitness/experience level could also be a possible explanation for the high variability in subject-specific model accuracies (Supplementary Tables S3, S5). For example, in the subject-specific NF vs. FT classifier, Participant 13 (VO_2_max = 58.2 ml/kg/min) had an accuracy of 50.2%, while Participant 10 (VO_2_max = 49.5 ml/kg/min) had an accuracy of 82.6%. The range was even wider in the subject-specific BEG vs. END classifier, with Participant 19 producing only a 36.4% accuracy, while Participant 22 produced a 94.7% accuracy. These ranges also may be due to differing individual strategies to manage fatigue, anthropometric characteristics, training approaches/history, injury status/history, etc. (41, 66, 67, 70–73), potential differences in day-to-day placement of commercial devices (74).

Irrespective of these limitations, the results of the current study emphasize how individual runners show different biomechanical responses to fatigue as compared to those reported in group-based models. These individual importance rankings showed salient differences from the group rankings, supporting the hypothesis that group-based information is likely insufficient to capture fatigue effects for most individuals nor to understand the intricacies of how fatiguing runs may alter biomechanical patterns. While inter-individual differences for certain variables have been previously noted, to our knowledge, this is the first to compare inter-individual differences across multiple variables in a single model.

More work is required before individualized running fatigue models could serve as the basis for an intervention protocol. Williams (13) advocated for identifying individuals' structural and functional abilities and determining how these abilities interact to influence performance and injury outcomes. Relevant variables to include in the models—as well as possible external influences on “typical” biomechanics, such as different footwear (75), listening to music (76), or mood (77)—would first have to be determined. Subsequently, the causes of these biomechanical alterations would have to be determined (e.g., weakness, asymmetries, pain, or other sources). Next, interventions could be designed to “correct” these issues and prolong non-fatigued biomechanics later into the run. However, based on the results presented here, it appears that a precision sports science approach is required to ultimately determine if fatigue-based interventions could be successful for injury prevention or performance enhancement.

There are other limitations in this study that must also be acknowledged. First, the devices used in Experiment 2 were commercial wearable devices with parameters derived from proprietary algorithms and were limited to the specific variables measured and calculated by the device. All devices had been previously lab validated and shown to be highly reliable, but additional variables important to the task (e.g., joint kinematics and kinetics) may have been missed (78). However, these devices are those that are typically used by runners to derive information about their training, and the complementary results between the two experiments emphasize the general underlying conclusions. Similarly, features selected from treadmill data were based on previous literature, and by making an *ad hoc* selection, relevant information could have been discarded (79). Second, in Experiment 2, external circumstances were not controlled and may have changed running form or influenced biomechanical adjustments in unknown ways. However, this also increases the real-world applicability of the analysis given the heterogeneity of circumstances most runners experience day-to-day. Third, some participants in Experiment 2 had longer runs than others, meaning that their END segments were further away from the BEG segment than others. This method was chosen to incorporate the segments that were most likely to represent a fatigued state. However, without knowing participant fitness or fatigue information, it is not clear which (if either) was the correct selection to demonstrate fatigue-related alterations in biomechanics. Fourth, there is a risk of overfitting given the relatively small sample sizes, particularly in the subject-specific model, although cross-validation procedures were employed to reduce this risk (80, 81). Finally, the sample sizes between the group-based and subject-specific models were not the same, which could impact the comparability of accuracy results, although it should not affect the interpretation of variable importance rankings. Additionally, by including minimum accuracies for significance (which are based on sample sizes), better performance comparisons may be made.

In conclusion, this study indicates that fatigue-related changes in running biomechanics are better described by subject-specific than group-based models. Additionally, this presents evidence that runners generally alter their biomechanics in a manner different from the aggregate, indicating that group-based models are likely insufficient to explain many individuals' responses to fatigue and thus unlikely to be the basis for successful intervention. In the future, adding more IMUs/additional types of sensors (e.g., heart rate, blood oxygenation) (78), using different IMU configurations (43), or applying different classifiers (27, 82) may improve accuracy and applicability. Researchers and developers will need to find the right balance of model type, sensor configuration, and measurement variables to make this type of system both efficacious and usable to coaches and clinicians.

## Data Availability

The raw data supporting the conclusions of this article will be made available by the authors, upon reasonable request.
