# Supplementary material for: Use of subject-specific models to detect fatigue-related changes in running biomechanics: a random forest approach
Source: Front Sports Act Living. 2023 Dec 21;5:1283316. doi: 10.3389/fspor.2023.1283316 (PMC10768007; doi:10.3389/fspor.2023.1283316)
Supplement: Supplementary file 1 [file Table1.docx]

| Classifier | F1 | Accuracy | Precision | Recall |
| --- | --- | --- | --- | --- |
| *Random Forest (used)* | *0.696 ± 0.165* | *0.689 ± 0.171* | *0.699 ± 0.166* | *0.707 ± 0.164* |
| Logistic Regression (LASSO) | 0.662 ± 0.162 | 0.668 ± 0.150 | 0.678 ± 0.131 | 0.680 ± 0.169 |
| Support Vector Machine | 0.670 ± 0.155 | 0.664 ± 0.161 | 0.645 ± 0.162 | 0.710 ± 0.135 |
| Naïve Bayes | 0.643 ± 0.184 | 0.642 ± 0.150 | 0.646 ± 0.142 | 0.716 ± 0.226 |

*Supplementary Table 1. Comparison of different classifiers from the subject-specific models for Experiment 2.*
