# Supplementary material for: Use of subject-specific models to detect fatigue-related changes in running biomechanics: a random forest approach
Source: Front Sports Act Living. 2023 Dec 21;5:1283316. doi: 10.3389/fspor.2023.1283316 (PMC10768007; doi:10.3389/fspor.2023.1283316)
Supplement: Supplementary file 2 [file Table2.docx]

| Classifier | F1 | Accuracy | Precision | Recall |
| --- | --- | --- | --- | --- |
| *Random Forest (used)* | *0.593 ± 0.174* | *0.615 ± 0.117* | *0.599 ± 0.089* | *0.616 ± 0.237* |
| Logistic Regression (LASSO) | 0.594 ± 0.143 | 0.613 ± 0.120 | 0.610 ± 0.117 | 0.589 ± 0.183 |
| Support Vector Machine | 0.599 ± 0.146 | 0.612 ± 0.112 | 0.624 ± 0.131 | 0.602 ± 0.205 |
| Naïve Bayes | 0.610 ± 0.185 | 0.520 ± 0.086 | 0.568 ± 0.173 | 0.873 ± 0.304 |

*Supplementary Table 2. Comparison of different classifiers from the group-based models for Experiment 2*
