# Supplementary material for: Use of subject-specific models to detect fatigue-related changes in running biomechanics: a random forest approach
Source: Front Sports Act Living. 2023 Dec 21;5:1283316. doi: 10.3389/fspor.2023.1283316 (PMC10768007; doi:10.3389/fspor.2023.1283316)
Supplement: Supplementary file 3 [file Table3.docx]

| Participant | Left-out Trial | Accuracy | F1 | Precision | Recall |
| --- | --- | --- | --- | --- | --- |
|  | 1 | 0.601 | 0.468 | 1.000 | 0.306 |
|  | 2 | 0.926 | 0.929 | 0.886 | 0.975 |
| 1 | 3 | 0.491 | 0.658 | 0.491 | 1.000 |
|  | 4 | 0.572 | 0.237 | 1.000 | 0.134 |
|  | **Mean** | **0.648** | **0.573** | **0.844** | **0.604** |
|  | **1** | 0.851 | 0.881 | 0.788 | 1.000 |
|  | 2 | 0.981 | 0.980 | 1.000 | 0.961 |
| 2 | 3 | 0.525 | 0.664 | 0.497 | 1.000 |
|  | 4 | 0.564 | 0.187 | 1.000 | 0.103 |
|  | **Mean** | **0.730** | **0.678** | **0.821** | **0.766** |
|  | 1 | 0.809 | 0.754 | 0.868 | 0.667 |
|  | **2** | 0.625 | 0.308 | 1.000 | 0.182 |
| 3 | 3 | 0.541 | 0.679 | 0.514 | 1.000 |
|  | 4 | 0.977 | 0.978 | 0.957 | 1.000 |
|  | **Mean** | **0.738** | **0.680** | **0.835** | **0.712** |
|  | 1 | 0.454 | 0.624 | 0.454 | 1.000 |
|  | 2 | 0.854 | 0.800 | 1.000 | 0.667 |
| 4 | **3** | 0.489 | 0.390 | 0.719 | 0.267 |
|  | 4 | 0.799 | 0.816 | 0.745 | 0.901 |
|  | **Mean** | **0.649** | **0.657** | **0.729** | **0.709** |
|  | 1 | 0.806 | 0.811 | 0.841 | 0.784 |
|  | 2 | 0.681 | 0.673 | 0.974 | 0.514 |
| 5 | 3 | 0.667 | 0.776 | 0.634 | 1.000 |
|  | **4** | 0.487 | 0.655 | 0.487 | 1.000 |
|  | **Mean** | **0.660** | **0.729** | **0.734** | **0.824** |
|  | 1 | 0.952 | 0.959 | 0.921 | 1.000 |
|  | 2 | 0.523 | 0.051 | 1.000 | 0.026 |
| 6 | 3 | 0.659 | 0.751 | 0.607 | 0.986 |
|  | 4 | 0.929 | 0.917 | 1.000 | 0.846 |
|  | **Mean** | **0.766** | **0.670** | **0.882** | **0.715** |
|  | 1 | 0.594 | 0.745 | 0.594 | 1.000 |
|  | 2 | 0.993 | 0.992 | 0.984 | 1.000 |
| 7 | 3 | 0.752 | 0.643 | 1.000 | 0.474 |
|  | 4 | 0.594 | 0.745 | 0.594 | 1.000 |
|  | **Mean** | **0.733** | **0.781** | **0.793** | **0.868** |
|  | **1** | 0.520 | 0.141 | 1.000 | 0.076 |
|  | 2 | 0.646 | 0.753 | 0.603 | 1.000 |
| 8 | 3 | 0.491 | 0.647 | 0.478 | 1.000 |
|  | 4 | 0.935 | 0.935 | 0.908 | 0.963 |
|  | **Mean** | **0.648** | **0.619** | **0.747** | **0.760** |
|  | 1 | 0.962 | 0.935 | 0.967 | 0.906 |
|  | **2** | 0.407 | 0.500 | 0.343 | 0.923 |
| 9 | 3 | 0.642 | 0.697 | 0.602 | 0.829 |
|  | 4 | 0.534 | 0.672 | 0.507 | 1.000 |
|  | **Mean** | **0.636** | **0.701** | **0.604** | **0.915** |
|  | 1 | 0.800 | 0.834 | 0.716 | 1.000 |
|  | 2 | 0.833 | 0.842 | 0.744 | 0.970 |
| 10 | **3** | 0.695 | 0.444 | 0.769 | 0.313 |
|  | 4 | 0.977 | 0.976 | 1.000 | 0.954 |
|  | **Mean** | **0.826** | **0.774** | **0.774** | **0.809** |
|  | 1 | 0.794 | 0.755 | 0.952 | 0.625 |
|  | 2 | 0.536 | 0.072 | 1.000 | 0.038 |
| 11 | 3 | 0.729 | 0.798 | 0.688 | 0.951 |
|  | **4** | 0.537 | 0.638 | 0.469 | 1.000 |
|  | **Mean** | **0.649** | **0.566** | **0.777** | **0.653** |
|  | 1 | 0.956 | 0.955 | 0.914 | 1.000 |
|  | 2 | 0.955 | 0.948 | 1.000 | 0.901 |
| 12 | 3 | 0.574 | 0.689 | 0.529 | 0.986 |
|  | 4 | 0.786 | 0.815 | 0.688 | 1.000 |
|  | **Mean** | **0.818** | **0.852** | **0.783** | **0.972** |
|  | 1 | 0.508 | 0.674 | 0.508 | 1.000 |
|  | 2 | 0.429 | 0.581 | 0.452 | 0.814 |
| 13 | 3 | 0.486 | 0.156 | 1.000 | 0.084 |
|  | 4 | 0.585 | 0.083 | 1.000 | 0.043 |
|  | **Mean** | **0.502** | **0.373** | **0.740** | **0.485** |
|  | 1 | 0.494 | 0.656 | 0.488 | 1.000 |
|  | 2 | 0.488 | 0.656 | 0.488 | 1.000 |
| 14 | 3 | 0.917 | 0.922 | 0.883 | 0.965 |
|  | 4 | 0.763 | 0.693 | 0.959 | 0.547 |
|  | **Mean** | **0.666** | **0.732** | **0.705** | **0.878** |
|  | 1 | 0.625 | 0.434 | 0.920 | 0.284 |
|  | 2 | 0.477 | 0.647 | 0.481 | 0.987 |
| 15 | 3 | 0.860 | 0.835 | 1.000 | 0.716 |
|  | 4 | 0.477 | 0.646 | 0.477 | 1.000 |
|  | **Mean** | **0.610** | **0.640** | **0.719** | **0.747** |
|  | 1 | 0.439 | 0.611 | 0.439 | 1.000 |
|  | 2 | 0.721 | 0.598 | 1.000 | 0.426 |
| 16 | 3 | 0.581 | 0.733 | 0.578 | 1.000 |
|  | 4 | 0.775 | 0.724 | 1.000 | 0.567 |
|  | **Mean** | **0.629** | **0.666** | **0.754** | **0.748** |

*Supplementary Table 3. Subject-specific random forest classifier details for Experiment 1.*
