# Supplementary material for: Use of subject-specific models to detect fatigue-related changes in running biomechanics: a random forest approach
Source: Front Sports Act Living. 2023 Dec 21;5:1283316. doi: 10.3389/fspor.2023.1283316 (PMC10768007; doi:10.3389/fspor.2023.1283316)
Supplement: Supplementary file 4 [file Table4.docx]

| Left-Out Participant | Accuracy | F1 | Precision | Recall |
| --- | --- | --- | --- | --- |
| 1 | 0.564 | 0.270 | 0.981 | 0.157 |
| 2 | 0.701 | 0.761 | 0.632 | 0.956 |
| 3 | 0.589 | 0.658 | 0.542 | 0.838 |
| 4 | 0.626 | 0.478 | 0.780 | 0.344 |
| 5 | 0.649 | 0.702 | 0.702 | 0.702 |
| 6 | 0.574 | 0.652 | 0.569 | 0.762 |
| 7 | 0.517 | 0.115 | 1.000 | 0.061 |
| 8 | 0.654 | 0.703 | 0.645 | 0.773 |
| 9 | 0.422 | 0.593 | 0.423 | 0.995 |
| 10 | 0.589 | 0.742 | 0.589 | 1.000 |
| 11 | 0.594 | 0.713 | 0.577 | 0.927 |
| 12 | 0.561 | 0.671 | 0.571 | 0.814 |
| 13 | 0.652 | 0.654 | 0.752 | 0.578 |
| 14 | 0.496 | 0.416 | 0.560 | 0.331 |
| 15 | 0.353 | 0.276 | 0.310 | 0.248 |
| 16 | 0.581 | 0.657 | 0.565 | 0.784 |
| **Mean** | **0.570** | **0.566** | **0.637** | **0.642** |

*Supplementary Table 4. Group-based random forest classifier details for Experiment 1.*
