# Supplementary material for: Use of subject-specific models to detect fatigue-related changes in running biomechanics: a random forest approach
Source: Front Sports Act Living. 2023 Dec 21;5:1283316. doi: 10.3389/fspor.2023.1283316 (PMC10768007; doi:10.3389/fspor.2023.1283316)
Supplement: Supplementary file 5 [file Table5.docx]

| Participant | Left-out Trial | Accuracy | F1 | Precision | Recall |
| --- | --- | --- | --- | --- | --- |
| 17 | 1 | 0.849 | 0.817 | 0.827 | 0.806 |
|  | 2 | 0.854 | 0.846 | 0.938 | 0.770 |
|  | 3 | 0.690 | 0.685 | 0.664 | 0.709 |
|  | 4 | 0.850 | 0.863 | 0.770 | 0.982 |
|  | 5 | 0.601 | 0.672 | 0.530 | 0.917 |
|  | **Mean** | **0.769** | **0.777** | **0.746** | **0.837** |
| 18 | 1 | 0.665 | 0.633 | 0.685 | 0.589 |
|  | 2 | 0.693 | 0.682 | 0.718 | 0.650 |
|  | 3 | 0.614 | 0.623 | 0.568 | 0.690 |
|  | 4 | 0.581 | 0.524 | 0.545 | 0.504 |
|  | 5 | 0.425 | 0.285 | 0.362 | 0.235 |
|  | **Mean** | **0.595** | **0.549** | **0.576** | **0.533** |
| 19 | 1 | 0.428 | 0.555 | 0.462 | 0.695 |
|  | 2 | 0.398 | 0.509 | 0.437 | 0.610 |
|  | 3 | 0.201 | 0.256 | 0.247 | 0.266 |
|  | 4 | 0.406 | 0.307 | 0.381 | 0.257 |
|  | 5 | 0.389 | 0.485 | 0.450 | 0.526 |
|  | **Mean** | **0.364** | **0.423** | **0.396** | **0.471** |
| 20 | 1 | 0.607 | 0.709 | 0.668 | 0.757 |
|  | 2 | 0.762 | 0.791 | 0.824 | 0.761 |
|  | 3 | 0.416 | 0.757 | 0.812 | 0.709 |
|  | 4 | 0.715 | 0.745 | 0.800 | 0.697 |
|  | 5 | 0.591 | 0.539 | 0.598 | 0.490 |
|  | **Mean** | **0.618** | **0.708** | **0.740** | **0.683** |
| 21 | 1 | 0.496 | 0.386 | 0.468 | 0.328 |
|  | 2 | 0.526 | 0.366 | 0.490 | 0.292 |
|  | 3 | 0.443 | 0.414 | 0.439 | 0.391 |
|  | 4 | 0.687 | 0.732 | 0.596 | 0.948 |
|  | 5 | 0.859 | 0.838 | 0.898 | 0.785 |
|  | **Mean** | **0.602** | **0.547** | **0.578** | **0.549** |
| 22 | 1 | 0.957 | 0.956 | 0.916 | 1.000 |
|  | 2 | 0.912 | 0.906 | 0.927 | 0.885 |
|  | 3 | 1.000 | 1.000 | 1.000 | 1.000 |
|  | 4 | 0.944 | 0.944 | 1.000 | 0.893 |
|  | 5 | 0.919 | 0.923 | 0.857 | 1.000 |
|  | **Mean** | **0.947** | **0.946** | **0.940** | **0.956** |

| 23 | 1 | 0.780 | 0.756 | 0.775 | 0.738 |
| --- | --- | --- | --- | --- | --- |
|  | 2 | 0.820 | 0.825 | 0.758 | 0.905 |
|  | 3 | 0.608 | 0.656 | 0.708 | 0.611 |
|  | 4 | 0.645 | 0.629 | 0.660 | 0.600 |
|  | 5 | 0.920 | 0.914 | 0.951 | 0.879 |
|  | **Mean** | **0.755** | **0.756** | **0.771** | **0.747** |
| 24 | 1 | 0.536 | 0.577 | 0.522 | 0.646 |
|  | 2 | 0.630 | 0.669 | 0.641 | 0.700 |
|  | 3 | 0.777 | 0.720 | 0.649 | 0.810 |
|  | 4 | 0.692 | 0.694 | 0.729 | 0.662 |
|  | 5 | 0.825 | 0.831 | 0.828 | 0.835 |
|  | **Mean** | **0.692** | **0.698** | **0.674** | **0.730** |
| 25 | 1 | 0.880 | 0.864 | 0.973 | 0.777 |
|  | 2 | 0.931 | 0.931 | 0.945 | 0.917 |
|  | 3 | 0.791 | 0.806 | 0.756 | 0.864 |
|  | 4 | 0.962 | 0.962 | 0.933 | 0.993 |
|  | 5 | 0.745 | 0.736 | 0.752 | 0.721 |
|  | **Mean** | **0.862** | **0.860** | **0.872** | **0.854** |

*Supplementary Table 5. Subject-specific random forest classifier details for Experiment 2.*
