# Supplementary material for: Use of subject-specific models to detect fatigue-related changes in running biomechanics: a random forest approach
Source: Front Sports Act Living. 2023 Dec 21;5:1283316. doi: 10.3389/fspor.2023.1283316 (PMC10768007; doi:10.3389/fspor.2023.1283316)
Supplement: Supplementary file 6 [file Table6.docx]

| Left-out Participant | Accuracy | F1 | Precision | Recall |
| --- | --- | --- | --- | --- |
| 17 | 0.671 | 0.670 | 0.629 | 0.716 |
| 18 | 0.511 | 0.482 | 0.493 | 0.472 |
| 19 | 0.529 | 0.571 | 0.543 | 0.602 |
| 20 | 0.440 | 0.243 | 0.493 | 0.161 |
| 21 | 0.675 | 0.681 | 0.636 | 0.733 |
| 22 | 0.744 | 0.784 | 0.666 | 0.952 |
| 23 | 0.531 | 0.475 | 0.541 | 0.423 |
| 24 | 0.644 | 0.640 | 0.622 | 0.659 |
| 25 | 0.788 | 0.795 | 0.765 | 0.827 |
| **Mean** | **0.615** | **0.593** | **0.599** | **0.616** |

*Supplementary Table 6. Group-based random forest classifier details for Experiment 2.*
