# Supplementary material for: Use of subject-specific models to detect fatigue-related changes in running biomechanics: a random forest approach
Source: Front Sports Act Living. 2023 Dec 21;5:1283316. doi: 10.3389/fspor.2023.1283316 (PMC10768007; doi:10.3389/fspor.2023.1283316)
Supplement: Supplementary file 7 [file Table7.docx]

| Partic. | Left-out Trial | Variable 1 | Variable 1 Imp. | Variable 2 | Variable 2 Imp. | Variable 3 | Variable 3 Imp. | Variable 4 | Variable 4 Imp. | Variable 5 | Variable 5 Imp. |
| --- | --- | --- | --- | --- | --- | --- | --- | --- | --- | --- | --- |
| 1 | 1 | P75 Z | 0.055 | SD R | 0.041 | MED Y | 0.038 | P25 Y | 0.038 | MEAN Y | 0.034 |
|  | 2 | MEAN Y | 0.043 | MEAN X | 0.041 | MEAN Z | 0.040 | MAX R | 0.040 | RMS Z | 0.033 |
|  | 3 | MAX R | 0.056 | RMS R | 0.047 | SD R | 0.046 | MAX Y | 0.046 | SE R | 0.040 |
|  | 4 | MEAN Z | 0.068 | MEAN X | 0.066 | P25 Y | 0.062 | SD R | 0.060 | P25 X | 0.047 |
| 2 | 1 | RATIO Y | 0.122 | SE Y | 0.080 | RMS Y | 0.078 | MEAN R | 0.076 | SD Y | 0.071 |
|  | 2 | SE Y | 0.083 | MIN Y | 0.078 | MED R | 0.074 | RMS Y | 0.058 | MEAN R | 0.048 |
|  | 3 | MIN Y | 0.130 | RMS Y | 0.074 | RATIO Y | 0.066 | SE Y | 0.062 | MEAN R | 0.056 |
|  | 4 | STD Y | 0.165 | RMS Y | 0.138 | RATIO Y | 0.114 | MEAN R | 0.089 | RMS R | 0.088 |
| 3 | 1 | P25 Z | 0.058 | MED Y | 0.052 | SD Z | 0.047 | MAX X | 0.044 | P75 R | 0.041 |
|  | 2 | P75 R | 0.045 | MAX Z | 0.041 | P75 X | 0.041 | SE R | 0.037 | MAX X | 0.037 |
|  | 3 | RATIO Z | 0.128 | SE X | 0.106 | SD Z | 0.096 | RMS Z | 0.062 | SE R | 0.051 |
|  | 4 | SE X | 0.085 | RATIO Z | 0.079 | MED Z | 0.074 | SD Z | 0.071 | RMS Z | 0.055 |
| 4 | 1 | SE X | 0.050 | P25 X | 0.045 | MEAN Y | 0.044 | MEAN Z | 0.042 | P75 X | 0.041 |
|  | 2 | P25 X | 0.053 | SE R | 0.045 | MEAN Z | 0.041 | P75 X | 0.039 | MAX R | 0.037 |
|  | 3 | SE R | 0.190 | SE X | 0.165 | P75 X | 0.123 | P25 X | 0.049 | P75 R | 0.033 |
|  | 4 | SE R | 0.158 | SE X | 0.132 | P25 X | 0.095 | P75 X | 0.069 | MEAN Y | 0.058 |
| 5 | 1 | SE R | 0.112 | MED X | 0.091 | SE X | 0.081 | SD Z | 0.066 | MAX X | 0.056 |
|  | 2 | MED X | 0.114 | RATIO X | 0.058 | SE R | 0.056 | SE X | 0.054 | P25 X | 0.052 |
|  | 3 | MED X | 0.177 | SE R | 0.090 | P25 X | 0.086 | MAX X | 0.067 | SE Z | 0.057 |
|  | 4 | SE X | 0.115 | MED X | 0.071 | SE Z | 0.061 | RATIO Z | 0.056 | SE R | 0.051 |
| 6 | 1 | SD Y | 0.085 | RMS Y | 0.083 | RATIO Y | 0.075 | SE Z | 0.071 | P25 X | 0.064 |
|  | 2 | RMS Y | 0.158 | SD Y | 0.154 | RATIO Y | 0.120 | MED R | 0.054 | P25 X | 0.053 |
|  | 3 | SE Z | 0.115 | RATIO Y | 0.105 | SD Y | 0.098 | RMS Y | 0.093 | RATIO Z | 0.065 |
|  | 4 | RMS Y | 0.046 | RATIO Y | 0.045 | P25 X | 0.045 | SE Z | 0.043 | SE X | 0.038 |
| 7 | 1 | MEAN R | 0.057 | P25 R | 0.056 | MED X | 0.056 | SD Z | 0.052 | MED R | 0.046 |
|  | 2 | MEAN R | 0.075 | P25 X | 0.074 | MAX X | 0.073 | P25 R | 0.059 | MED R | 0.043 |
|  | 3 | P25 R | 0.052 | RMS X | 0.046 | MEAN R | 0.045 | MEAN Y | 0.041 | MED X | 0.041 |
|  | 4 | P25 R | 0.066 | MED X | 0.059 | MED R | 0.057 | MEAN R | 0.042 | SD Z | 0.040 |
| 8 | 1 | P25 R | 0.158 | MEAN X | 0.096 | SE Y | 0.066 | MAX Z | 0.056 | MEAN Z | 0.055 |
|  | 2 | SE Y | 0.093 | P25 R | 0.081 | MIN Z | 0.065 | MEAN X | 0.059 | SE R | 0.058 |
|  | 3 | P25 R | 0.045 | MEAN X | 0.045 | SE X | 0.039 | SE Y | 0.035 | MEAN R | 0.035 |
|  | 4 | P25 R | 0.110 | MIN Z | 0.066 | P75 R | 0.066 | SE R | 0.057 | MED X | 0.055 |
| 9 | 1 | RMS Z | 0.054 | RATIO Z | 0.054 | RATIO X | 0.045 | SE Z | 0.045 | P75 X | 0.038 |
|  | 2 | RMS Z | 0.185 | RATIO X | 0.110 | RATIO Z | 0.103 | SE Z | 0.076 | MAX R | 0.051 |
|  | 3 | SE Z | 0.102 | RATIO Z | 0.095 | P75 Z | 0.055 | P25 X | 0.045 | RMS X | 0.044 |
|  | 4 | RATIO Z | 0.136 | RMS Z | 0.076 | P25 X | 0.062 | P75 X | 0.046 | RMS X | 0.046 |
| 10 | 1 | SE Z | 0.139 | RATIO Z | 0.091 | MEAN R | 0.080 | P25 R | 0.078 | RMS Z | 0.070 |
|  | 2 | SE Z | 0.108 | RATIO Z | 0.092 | P25 Y | 0.054 | P25 R | 0.053 | SD Y | 0.051 |
|  | 3 | SE Z | 0.234 | P25 Y | 0.099 | RATIO Z | 0.090 | SD Y | 0.060 | MEAN R | 0.051 |
|  | 4 | SE Z | 0.158 | SE X | 0.077 | RATIO Z | 0.060 | MEAN R | 0.052 | SE R | 0.052 |
| 11 | 1 | SD X | 0.050 | MAX R | 0.043 | SD R | 0.042 | MAX X | 0.038 | RMS Z | 0.037 |
|  | 2 | SD X | 0.064 | MIN Z | 0.046 | SD Z | 0.046 | RMS X | 0.046 | RMS R | 0.043 |
|  | 3 | MED Y | 0.125 | SD R | 0.083 | SD Z | 0.068 | SD X | 0.054 | P75 R | 0.052 |
|  | 4 | SD R | 0.060 | RMS R | 0.052 | MAX X | 0.045 | SD X | 0.041 | MIN Z | 0.041 |
| 12 | 1 | SE X | 0.133 | SE R | 0.125 | MAX X | 0.109 | SE Z | 0.085 | MIN Y | 0.067 |
|  | 2 | SE R | 0.142 | MAX X | 0.107 | MED R | 0.084 | SE X | 0.081 | MIN Y | 0.070 |
|  | 3 | SE R | 0.054 | MAX X | 0.044 | P75 R | 0.043 | SE X | 0.040 | SD Z | 0.039 |
|  | 4 | SE R | 0.282 | SE X | 0.187 | MAX X | 0.098 | MED X | 0.069 | P75 X | 0.047 |
| 13 | 1 | SE Y | 0.043 | RATIO X | 0.041 | SD Z | 0.039 | SE R | 0.037 | MAX R | 0.035 |
|  | 2 | SE Z | 0.075 | P25 X | 0.070 | MAX X | 0.061 | SE R | 0.053 | P25 Z | 0.052 |
|  | 3 | RMS R | 0.130 | MAX X | 0.116 | MEAN R | 0.087 | RATIO X | 0.082 | SE R | 0.050 |
|  | 4 | MEAN R | 0.127 | RMS R | 0.085 | RATIO Z | 0.080 | RMS Y | 0.055 | MIN Y | 0.052 |
| 14 | 1 | MED R | 0.044 | RMS Y | 0.043 | MED X | 0.042 | P75 X | 0.041 | MEAN Y | 0.040 |
|  | 2 | SD Y | 0.069 | RATIO Z | 0.047 | RATIO Y | 0.045 | P75 X | 0.044 | RMS R | 0.039 |
|  | 3 | P75 X | 0.103 | RATIO X | 0.074 | SE Y | 0.068 | RATIO Y | 0.063 | P75 R | 0.062 |
|  | 4 | P75 X | 0.154 | RATIO Y | 0.098 | SD Y | 0.090 | RMS Y | 0.080 | MED X | 0.062 |
| 15 | 1 | P25 X | 0.093 | MAX R | 0.071 | MAX X | 0.065 | MED Z | 0.050 | MEAN R | 0.049 |
|  | 2 | P25 X | 0.127 | SD X | 0.067 | RMS X | 0.054 | P25 R | 0.050 | RMS Z | 0.038 |
|  | 3 | P25 X | 0.101 | RATIO Y | 0.082 | MEAN R | 0.065 | P25 R | 0.054 | MIN Z | 0.047 |
|  | 4 | MAX R | 0.115 | P25 X | 0.092 | MAX X | 0.063 | RMS X | 0.058 | P25 R | 0.053 |
| 16 | 1 | SE Y | 0.093 | MED Z | 0.093 | MEAN Z | 0.069 | RMS R | 0.052 | P25 X | 0.045 |
|  | 2 | SE Y | 0.182 | RMS Y | 0.058 | SD Z | 0.058 | SD Y | 0.057 | P25 Z | 0.052 |
|  | 3 | P25 X | 0.046 | P75 R | 0.045 | RMS Y | 0.042 | MED Z | 0.040 | P75 X | 0.038 |
|  | 4 | SE Y | 0.053 | RMS R | 0.053 | P25 X | 0.052 | MEAN Z | 0.047 | SD X | 0.040 |

| Partic. | Left-out Trial | Variable 6 | Variable 6 Imp. | Variable 7 | Variable 7 Imp. | Variable 8 | Variable 8 Imp. | Variable 9 | Variable 9 Imp. | Variable 10 | Variable 10 Imp. |
| --- | --- | --- | --- | --- | --- | --- | --- | --- | --- | --- | --- |
| 1 | 1 | P25 X | 0.032 | SE X | 0.032 | SD Z | 0.031 | MAX Y | 0.031 | RATIO Z | 0.031 |
|  | 2 | MED Y | 0.033 | SD R | 0.032 | P75 Z | 0.032 | MIN R | 0.032 | MIN Z | 0.031 |
|  | 3 | SE Z | 0.039 | SD Y | 0.039 | RMS Y | 0.038 | MAX X | 0.038 | MED Z | 0.038 |
|  | 4 | RATIO Z | 0.047 | MIN Z | 0.035 | MED Z | 0.034 | SD Z | 0.033 | RMS Z | 0.032 |
| 2 | 1 | MIN Y | 0.064 | RMS R | 0.052 | P25 X | 0.048 | P75 X | 0.044 | MAX Y | 0.037 |
|  | 2 | MED X | 0.047 | SD Y | 0.047 | P75 X | 0.047 | RMS R | 0.039 | P25 X | 0.035 |
|  | 3 | SD Y | 0.056 | RATIO X | 0.046 | MIN Z | 0.040 | MAX R | 0.038 | RMS R | 0.038 |
|  | 4 | MIN Y | 0.079 | P25 X | 0.054 | MED R | 0.042 | P75 X | 0.026 | SE Y | 0.024 |
| 3 | 1 | MED R | 0.039 | MED X | 0.038 | SE Z | 0.038 | RMS Z | 0.037 | SE X | 0.037 |
|  | 2 | MED Y | 0.035 | SE X | 0.035 | P25 Y | 0.034 | RATIO Z | 0.031 | MEAN Y | 0.031 |
|  | 3 | P75 X | 0.043 | MED Y | 0.042 | SE Z | 0.041 | SD X | 0.034 | P75 R | 0.033 |
|  | 4 | P75 Y | 0.046 | MED Y | 0.044 | RATIO X | 0.041 | P75 R | 0.035 | P75 X | 0.034 |
| 4 | 1 | RMS Y | 0.039 | P75 R | 0.039 | P75 Y | 0.037 | SD Z | 0.035 | SE R | 0.033 |
|  | 2 | SD Z | 0.036 | SE X | 0.035 | P25 Z | 0.034 | SD X | 0.034 | MIN Y | 0.033 |
|  | 3 | MEAN Z | 0.029 | RATIO Z | 0.028 | P75 Y | 0.026 | P25 Z | 0.026 | P75 Z | 0.025 |
|  | 4 | P75 Y | 0.047 | MEAN Z | 0.046 | P75 Z | 0.041 | RATIO X | 0.033 | RATIO Z | 0.029 |
| 5 | 1 | RATIO Z | 0.049 | MAX Z | 0.049 | MED R | 0.046 | P75 Y | 0.040 | RATIO X | 0.038 |
|  | 2 | RATIO Z | 0.050 | RMS Z | 0.048 | P75 R | 0.048 | SE Z | 0.041 | MAX Y | 0.039 |
|  | 3 | MED Z | 0.051 | MAX R | 0.040 | SE Y | 0.040 | RATIO Z | 0.033 | P75 Y | 0.031 |
|  | 4 | MIN Z | 0.051 | RATIO X | 0.046 | MED R | 0.045 | RMS Z | 0.036 | MAX X | 0.036 |
| 6 | 1 | SE X | 0.055 | MAX R | 0.050 | P75 X | 0.046 | P75 Y | 0.039 | RAITO Z | 0.038 |
|  | 2 | P25 Y | 0.047 | P75 Y | 0.039 | P25 Z | 0.036 | SD R | 0.030 | RMS R | 0.029 |
|  | 3 | P25 X | 0.058 | SE X | 0.053 | SE Y | 0.049 | P75 X | 0.039 | SD R | 0.034 |
|  | 4 | P25 Z | 0.037 | SD Y | 0.035 | RMS R | 0.034 | MAX R | 0.032 | MAX X | 0.032 |
| 7 | 1 | RMS Z | 0.043 | P75 Z | 0.038 | MIN X | 0.033 | P25 X | 0.032 | MIN Z | 0.030 |
|  | 2 | MAX R | 0.039 | MIN Y | 0.037 | RATIO X | 0.037 | MIN X | 0.037 | RMS R | 0.035 |
|  | 3 | MIN X | 0.039 | RMS R | 0.038 | SD X | 0.035 | P25 X | 0.033 | SE X | 0.032 |
|  | 4 | MIN X | 0.038 | MEAN Y | 0.033 | MED Z | 0.032 | P75 Y | 0.032 | RMS X | 0.031 |
| 8 | 1 | P25 Y | 0.052 | SE X | 0.038 | MED X | 0.037 | MIN Z | 0.035 | MEAN R | 0.031 |
|  | 2 | SD Y | 0.057 | MEAN R | 0.056 | MEAN Z | 0.052 | MAX Z | 0.049 | RATIO Y | 0.046 |
|  | 3 | MAX Z | 0.032 | MEAN Z | 0.032 | MIN Z | 0.031 | P25 Y | 0.031 | P75 R | 0.029 |
|  | 4 | MEAN Z | 0.054 | SE Y | 0.051 | MEAN X | 0.051 | MAX Z | 0.045 | P25 X | 0.045 |
| 9 | 1 | SD Z | 0.037 | RMS X | 0.035 | SE R | 0.034 | P25 X | 0.033 | MEAN Y | 0.032 |
|  | 2 | SE R | 0.049 | P75 X | 0.049 | MAX X | 0.035 | SD Z | 0.035 | P25 X | 0.030 |
|  | 3 | SD Z | 0.037 | SD X | 0.036 | RMS Z | 0.035 | SE X | 0.035 | P75 X | 0.033 |
|  | 4 | MEAN Y | 0.045 | SE R | 0.037 | SD X | 0.035 | SE Z | 0.030 | SE X | 0.029 |
| 10 | 1 | P25 Z | 0.055 | MEAN Z | 0.034 | MED R | 0.032 | MED Z | 0.032 | P75 R | 0.030 |
|  | 2 | RMS Z | 0.046 | SE R | 0.044 | SD Z | 0.039 | MEAN R | 0.038 | RMS Y | 0.038 |
|  | 3 | MIN X | 0.046 | RMS Y | 0.043 | P25 R | 0.040 | P75 Y | 0.039 | RMS Z | 0.038 |
|  | 4 | MED R | 0.047 | P25 Z | 0.045 | P25 R | 0.039 | RMS R | 0.030 | MED Z | 0.027 |
| 11 | 1 | RMS R | 0.035 | SD Y | 0.034 | P25 Y | 0.033 | MIN Z | 0.032 | RATIO X | 0.031 |
|  | 2 | SD R | 0.042 | P75 R | 0.041 | RMS Z | 0.040 | SE X | 0.038 | P75 X | 0.038 |
|  | 3 | RMS Z | 0.052 | RMS R | 0.045 | P25 Y | 0.044 | RMS X | 0.036 | P75 X | 0.033 |
|  | 4 | SE Z | 0.038 | RMS Z | 0.036 | RATIO Z | 0.036 | RMS X | 0.036 | SD X | 0.032 |
| 12 | 1 | P75 R | 0.039 | RMS Y | 0.037 | MED Y | 0.036 | SD Y | 0.028 | RATIO Y | 0.022 |
|  | 2 | MEAN R | 0.057 | P75 X | 0.046 | MED X | 0.045 | P75 R | 0.041 | MED Z | 0.031 |
|  | 3 | MEAN R | 0.036 | MEAN X | 0.034 | RATIO X | 0.033 | MIN R | 0.032 | P75 X | 0.031 |
|  | 4 | MED Y | 0.035 | MEAN R | 0.034 | MED R | 0.024 | RMS Y | 0.019 | P75 R | 0.018 |
| 13 | 1 | MIN Y | 0.035 | MAX X | 0.034 | MIN Z | 0.034 | SD Y | 0.033 | MEAN Z | 0.032 |
|  | 2 | SD X | 0.051 | MAX R | 0.049 | RMS Y | 0.048 | SE Y | 0.046 | MEAN Y | 0.043 |
|  | 3 | SD R | 0.044 | MAX R | 0.042 | SE Y | 0.041 | MIN Y | 0.039 | RMS Y | 0.033 |
|  | 4 | P25 X | 0.051 | SD Y | 0.050 | RMS Z | 0.046 | SE X | 0.045 | RATIO X | 0.040 |
| 14 | 1 | SE Y | 0.038 | RATIO Y | 0.036 | SD Y | 0.035 | RATIO X | 0.034 | P25 X | 0.032 |
|  | 2 | SE Y | 0.038 | SE Z | 0.036 | RMS Y | 0.035 | MED X | 0.033 | P75 R | 0.032 |
|  | 3 | MED X | 0.049 | P25 X | 0.047 | SD Y | 0.043 | RMS Y | 0.043 | P25 R | 0.038 |
|  | 4 | P75 R | 0.044 | SE Y | 0.044 | MEAN Y | 0.041 | MED R | 0.041 | P75 Z | 0.041 |
| 15 | 1 | SE Y | 0.049 | SE Z | 0.047 | SD R | 0.044 | P25 R | 0.039 | RATIO Y | 0.036 |
|  | 2 | RATIO Y | 0.038 | SE Z | 0.037 | RMS R | 0.037 | SE R | 0.036 | P25 Z | 0.034 |
|  | 3 | SE Y | 0.046 | MEAN Z | 0.044 | SE Z | 0.043 | P75 X | 0.043 | SD Y | 0.042 |
|  | 4 | SE Y | 0.052 | RATIO Y | 0.041 | RMS R | 0.036 | MEAN Y | 0.036 | SD X | 0.035 |
| 16 | 1 | MEAN Y | 0.042 | SD Y | 0.042 | RMS X | 0.037 | SD R | 0.033 | SD X | 0.032 |
|  | 2 | SD R | 0.048 | MAX R | 0.046 | P25 X | 0.044 | MIN Z | 0.043 | RMS R | 0.042 |
|  | 3 | SD Z | 0.035 | SE Y | 0.035 | RMS R | 0.034 | SD R | 0.034 | MAX R | 0.033 |
|  | 4 | SD R | 0.040 | P75 R | 0.039 | MED Z | 0.032 | MEAN R | 0.031 | MAX R | 0.031 |

| Partic. | Left-out Trial | Variable 11 | Variable 11 Imp. | Variable 12 | Variable 12 Imp. | Variable 13 | Variable 13 Imp. | Variable 14 | Variable 14 Imp. | Variable 15 | Variable 15 Imp. |
| --- | --- | --- | --- | --- | --- | --- | --- | --- | --- | --- | --- |
| 1 | 1 | MEAN X | 0.030 | RMS R | 0.030 | RATIO X | 0.028 | MEAN Z | 0.028 | MIN R | 0.028 |
|  | 2 | MED Z | 0.030 | MAX Y | 0.029 | RATIO Z | 0.029 | MED X | 0.028 | SE X | 0.028 |
|  | 3 | MEAN Z | 0.034 | RMS Z | 0.031 | SE X | 0.031 | MAX Z | 0.029 | SD Z | 0.025 |
|  | 4 | MEAN Y | 0.029 | RATIO X | 0.027 | SE X | 0.027 | MED Y | 0.026 | MAX Z | 0.025 |
| 2 | 1 | P75 R | 0.027 | MAX R | 0.026 | MED X | 0.026 | P25 R | 0.026 | MED R | 0.021 |
|  | 2 | P25 R | 0.030 | SE R | 0.029 | RATIO X | 0.029 | RATIO Y | 0.028 | MAX Y | 0.028 |
|  | 3 | P75 X | 0.025 | MED R | 0.024 | SE R | 0.024 | P75 R | 0.024 | SD Z | 0.023 |
|  | 4 | P25 R | 0.023 | MED X | 0.019 | P25 Y | 0.015 | P75 R | 0.015 | MAX R | 0.013 |
| 3 | 1 | P75 X | 0.036 | P25 Y | 0.030 | MAX Z | 0.030 | SD R | 0.028 | RATIO Z | 0.027 |
|  | 2 | SE Z | 0.031 | MED X | 0.031 | SD X | 0.030 | P25 Z | 0.029 | SD Z | 0.029 |
|  | 3 | RMS X | 0.031 | P25 Z | 0.027 | MEAN Z | 0.025 | MEAN Y | 0.022 | RMS R | 0.022 |
|  | 4 | SE R | 0.034 | P25 Y | 0.033 | SE Z | 0.033 | MED X | 0.026 | P25 Z | 0.026 |
| 4 | 1 | P75 Z | 0.031 | MED Z | 0.029 | SD Y | 0.029 | RMS R | 0.026 | MED X | 0.025 |
|  | 2 | MED Z | 0.032 | RMS Z | 0.030 | MAX X | 0.030 | MED R | 0.030 | RATIO X | 0.028 |
|  | 3 | MED Z | 0.024 | SD Z | 0.023 | MIN Y | 0.021 | RATIO Y | 0.020 | RMS Z | 0.019 |
|  | 4 | MAX X | 0.027 | SD Z | 0.027 | P25 Z | 0.027 | MED Z | 0.024 | MAX R | 0.022 |
| 5 | 1 | MAX Y | 0.037 | P25 X | 0.032 | RMS Y | 0.032 | MAX R | 0.030 | RMS Z | 0.019 |
|  | 2 | MED Z | 0.033 | MAX X | 0.033 | MAX R | 0.031 | SD Z | 0.026 | SD Y | 0.023 |
|  | 3 | MED R | 0.031 | RATIO Y | 0.030 | MEAN Z | 0.029 | MAX Z | 0.023 | RMS Z | 0.021 |
|  | 4 | P75 R | 0.034 | P75 Y | 0.033 | SE Y | 0.033 | P25 X | 0.028 | MAX Y | 0.025 |
| 6 | 1 | MAX X | 0.034 | P25 R | 0.033 | P25 Y | 0.031 | P25 Z | 0.028 | MED R | 0.026 |
|  | 2 | SE Y | 0.026 | MAX X | 0.022 | MAX R | 0.022 | MED Z | 0.022 | SE X | 0.020 |
|  | 3 | MED R | 0.030 | P25 R | 0.024 | MEAN Y | 0.022 | P25 Z | 0.021 | P25 Y | 0.018 |
|  | 4 | P75 X | 0.030 | MEAN Y | 0.030 | MED R | 0.029 | MAX Z | 0.029 | MED Z | 0.027 |
| 7 | 1 | P75 Y | 0.030 | RATIO Z | 0.029 | MED Z | 0.026 | MED Y | 0.025 | MAX X | 0.025 |
|  | 2 | RMS Z | 0.034 | P75 X | 0.031 | RMS X | 0.028 | P25 Y | 0.027 | SD Z | 0.027 |
|  | 3 | MED R | 0.031 | SD Z | 0.031 | MEAN Z | 0.031 | MAX X | 0.028 | RATIO Z | 0.028 |
|  | 4 | MAX R | 0.031 | RATIO Z | 0.030 | MIN R | 0.030 | SD X | 0.028 | RMS Z | 0.028 |
| 8 | 1 | P25 X | 0.027 | MED Y | 0.026 | P75 Y | 0.026 | P75 Z | 0.026 | MEAN Y | 0.022 |
|  | 2 | SE X | 0.043 | P25 Y | 0.033 | MIN Y | 0.030 | MED Y | 0.029 | MED R | 0.026 |
|  | 3 | MED Y | 0.028 | SD R | 0.027 | SE R | 0.027 | RATIO Y | 0.027 | P75 Y | 0.026 |
|  | 4 | SE X | 0.035 | P75 Y | 0.031 | RATIO Y | 0.029 | MED Y | 0.027 | P25 Y | 0.024 |
| 9 | 1 | MED Z | 0.030 | MAX X | 0.029 | MAX R | 0.029 | MAX Y | 0.028 | SE X | 0.027 |
|  | 2 | P75 Z | 0.030 | MAX Y | 0.023 | SE X | 0.017 | P25 R | 0.017 | MEAN Y | 0.017 |
|  | 3 | SD R | 0.029 | MAX R | 0.028 | SE R | 0.028 | MAX X | 0.026 | P25 R | 0.025 |
|  | 4 | RATIO X | 0.029 | SD Z | 0.029 | MIN Z | 0.027 | MAX R | 0.025 | P75 Z | 0.024 |
| 10 | 1 | P75 Y | 0.030 | SD Y | 0.029 | MIN X | 0.027 | P25 Y | 0.023 | RMS Y | 0.020 |
|  | 2 | RATIO Y | 0.038 | P75 Y | 0.037 | MEAN X | 0.028 | MIN X | 0.026 | RMS R | 0.025 |
|  | 3 | RMS X | 0.031 | SD X | 0.028 | MED R | 0.028 | RATIO Y | 0.024 | P75 R | 0.017 |
|  | 4 | MAX X | 0.026 | P25 Y | 0.026 | P75 R | 0.026 | MEAN Z | 0.022 | RMS Z | 0.021 |
| 11 | 1 | SD Z | 0.031 | RMS X | 0.030 | MED Y | 0.029 | SE Z | 0.028 | SE X | 0.028 |
|  | 2 | MAX R | 0.035 | P25 Y | 0.029 | SE Z | 0.028 | P25 Z | 0.027 | MED R | 0.027 |
|  | 3 | MEAN R | 0.032 | RATIO X | 0.032 | SE Z | 0.031 | RATIO Z | 0.029 | P25 X | 0.029 |
|  | 4 | P25 Y | 0.030 | SD Y | 0.028 | MEAN Z | 0.027 | MIN Y | 0.026 | MED Y | 0.025 |
| 12 | 1 | MAX Y | 0.022 | MED R | 0.022 | MAX R | 0.021 | MEAN X | 0.021 | MED X | 0.020 |
|  | 2 | SD Y | 0.031 | MED Y | 0.028 | RMS Y | 0.026 | RMS R | 0.023 | MEAN X | 0.020 |
|  | 3 | MED R | 0.031 | RATIO Y | 0.030 | RMS R | 0.029 | SE Z | 0.028 | SD Y | 0.027 |
|  | 4 | MIN Y | 0.013 | MEAN X | 0.012 | SD Y | 0.011 | MED Z | 0.011 | MEAN Y | 0.011 |
| 13 | 1 | SD R | 0.032 | MEAN X | 0.031 | RATIO Z | 0.028 | SE Z | 0.027 | SD X | 0.027 |
|  | 2 | SD Y | 0.039 | RMS X | 0.038 | RMS R | 0.032 | MAX Z | 0.031 | SE X | 0.023 |
|  | 3 | SD Y | 0.033 | SE X | 0.031 | RMS Z | 0.026 | P25 X | 0.026 | RMS X | 0.024 |
|  | 4 | MIN Z | 0.038 | SD Z | 0.038 | SE Y | 0.037 | SD X | 0.036 | MAX X | 0.033 |
| 14 | 1 | RATIO Z | 0.032 | SD X | 0.032 | SE R | 0.032 | SD Z | 0.030 | MIN R | 0.028 |
|  | 2 | MAX R | 0.030 | RMS Z | 0.029 | SD R | 0.028 | SD Z | 0.028 | MED X | 0.025 |
|  | 3 | SD R | 0.029 | SE Z | 0.028 | MEAN Y | 0.028 | P75 Z | 0.027 | RMS R | 0.024 |
|  | 4 | P25 X | 0.032 | RATIO X | 0.031 | SE Z | 0.024 | MIN R | 0.020 | RATIO Z | 0.018 |
| 15 | 1 | MIN Z | 0.034 | P25 Z | 0.031 | RMS Z | 0.030 | SD Y | 0.028 | SD X | 0.027 |
|  | 2 | MAX X | 0.033 | SD R | 0.033 | MAX R | 0.033 | MIN Z | 0.032 | MEAN Z | 0.032 |
|  | 3 | MEAN Y | 0.037 | MED Z | 0.036 | MAX R | 0.030 | P25 Z | 0.026 | RMS Y | 0.023 |
|  | 4 | P75 X | 0.033 | MEAN R | 0.032 | MEAN Z | 0.031 | SD Y | 0.029 | RMS Y | 0.028 |
| 16 | 1 | SD Z | 0.031 | P75 X | 0.031 | P75 R | 0.028 | RATIO Z | 0.028 | MAX X | 0.028 |
|  | 2 | MED Z | 0.040 | MEAN Z | 0.039 | SD X | 0.029 | MEAN Y | 0.025 | SE R | 0.025 |
|  | 3 | MEAN Z | 0.032 | P25 Z | 0.030 | SD Y | 0.030 | MAX Z | 0.030 | RMS X | 0.028 |
|  | 4 | MAX Z | 0.029 | SD Y | 0.028 | P75 Y | 0.028 | P25 R | 0.027 | SE X | 0.025 |

| Partic. | Left-out Trial | Variable 16 | Variable 16 Imp. | Variable 17 | Variable 17 Imp. | Variable 18 | Variable 18 Imp. | Variable 19 | Variable 19 Imp. | Variable 20 | Variable 20 Imp. |
| --- | --- | --- | --- | --- | --- | --- | --- | --- | --- | --- | --- |
| 1 | 1 | P25 R | 0.027 | RMS Y | 0.025 | MAX R | 0.024 | SE Y | 0.023 | P25 Z | 0.23 |
|  | 2 | RATIO X | 0.027 | MAX X | 0.027 | P25 X | 0.027 | P25 Y | 0.026 | MIN Y | 0.025 |
|  | 3 | MIN Z | 0.024 | MIN R | 0.023 | RMS X | 0.023 | SE Y | 0.022 | RATIO Z | 0.022 |
|  | 4 | SE Y | 0.023 | P75 X | 0.023 | MED R | 0.021 | MED X | 0.019 | RMS R | 0.019 |
| 2 | 1 | RATIO X | 0.021 | SD R | 0.019 | MIN R | 0.019 | MEAN Z | 0.019 | P25 Y | 0.017 |
|  | 2 | P75 R | 0.026 | MAX R | 0.024 | MAX X | 0.024 | SD X | 0.019 | MEAN Z | 0.017 |
|  | 3 | MEAN Y | 0.020 | MAX Y | 0.020 | SD X | 0.019 | MED X | 0.018 | MEAN Z | 0.016 |
|  | 4 | MIN Z | 0.013 | SD X | 0.012 | MAX Y | 0.11 | MIN R | 0.009 | RATIO X | 0.006 |
| 3 | 1 | MED Z | 0.026 | P75 Y | 0.026 | SD X | 0.025 | P25 X | 0.024 | MIN X | 0.024 |
|  | 2 | P75 Y | 0.028 | MED R | 0.028 | P25 R | 0.027 | RMS X | 0.026 | MIN X | 0.025 |
|  | 3 | RATIO X | 0.019 | MAX X | 0.017 | MEAN R | 0.016 | MIN Z | 0.016 | MAX Y | 0.016 |
|  | 4 | MAX Z | 0.026 | SD X | 0.022 | MEAN Y | 0.21 | MED R | 0.017 | RMS X | 0.016 |
| 4 | 1 | P25 R | 0.025 | SE Y | 0.024 | MEAN R | 0.024 | P25 Z | 0.023 | MED R | 0.022 |
|  | 2 | RMS X | 0.026 | P75 Y | 0.025 | RATIO Y | 0.024 | SD Y | 0.023 | MIN R | 0.023 |
|  | 3 | RATIO X | 0.019 | MEAN Y | 0.018 | RMS Y | 0.017 | RMS R | 0.016 | MED R | 0.014 |
|  | 4 | SD R | 0.018 | RATIO Y | 0.013 | MED Y | 0.012 | RMS Z | 0.012 | MED R | 0.012 |
| 5 | 1 | SE Y | 0.018 | MIN Y | 0.018 | SD Y | 0.018 | MED Z | 0.016 | P75 R | 0.015 |
|  | 2 | MED Y | 0.022 | P75 Y | 0.020 | MEAN Z | 0.020 | MED R | 0.019 | MIN Z | 0.019 |
|  | 3 | P75 R | 0.020 | RMS Y | 0.018 | MAX Y | 0.018 | MIN Z | 0.018 | SD Y | 0.015 |
|  | 4 | MEAN Z | 0.025 | SD Z | 0.022 | MAX R | 0.019 | MIN X | 0.017 | MED Y | 0.016 |
| 6 | 1 | MEAN Y | 0.023 | MED Z | 0.022 | MAX Z | 0.019 | SD R | 0.016 | RMS Z | 0.016 |
|  | 2 | MED X | 0.018 | MAX Y | 0.015 | RATIO Z | 0.012 | P25 R | 0.010 | RATIO X | 0.009 |
|  | 3 | RMS Z | 0.015 | MAX Z | 0.014 | MAX X | 0.014 | P75 Z | 0.013 | P75 Y | 0.013 |
|  | 4 | RATIO Z | 0.025 | P25 Y | 0.025 | P75 Y | 0.025 | SD R | 0.025 | MED X | 0.24 |
| 7 | 1 | SD X | 0.025 | RMS R | 0.025 | P25 Y | 0.022 | MAX R | 0.022 | P75 X | 0.021 |
|  | 2 | MEAN Y | 0.026 | MIN R | 0.025 | RATIO Z | 0.024 | MED X | 0.023 | MIN Z | 0.022 |
|  | 3 | RATIO X | 0.027 | MED Z | 0.027 | MIN R | 0.026 | P75 X | 0.024 | P75 Z | 0.024 |
|  | 4 | RMS R | 0.027 | MIN Z | 0.027 | P25 X | 0.025 | RATIO X | 0.025 | MAX X | 0.024 |
| 8 | 1 | SE Z | 0.020 | P75 X | 0.019 | MAX Y | 0.019 | SE R | 0.016 | RATIO Z | 0.014 |
|  | 2 | RMS Y | 0.025 | MEAN Y | 0.023 | P25 X | 0.020 | P75 Y | 0.019 | RATIO X | 0.015 |
|  | 3 | MIN Y | 0.026 | SD Z | 0.026 | RATIO Z | 0.026 | P25 Z | 0.026 | MED X | 0.025 |
|  | 4 | MEAN R | 0.021 | MEAN Y | 0.020 | RMS Y | 0.018 | P75 Z | 0.018 | MAX R | 0.016 |
| 9 | 1 | MEAN Z | 0.027 | P75 Z | 0.025 | SD X | 0.024 | MIN Z | 0.023 | MED X | 0.023 |
|  | 2 | P75 Y | 0.016 | MED X | 0.014 | MED Z | 0.013 | SD X | 0.013 | RMS X | 0.013 |
|  | 3 | MED Z | 0.025 | RATIO X | 0.024 | RMS R | 0.024 | MED Y | 0.022 | MED R | 0.021 |
|  | 4 | P75 Y | 0.024 | MED Z | 0.022 | MAX Y | 0.021 | SE Y | 0.021 | MEAN Z | 0.019 |
| 10 | 1 | MED Y | 0.020 | RMS R | 0.018 | RMS X | 0.018 | SE R | 0.018 | SD X | 0.018 |
|  | 2 | SD X | 0.020 | P25 Z | 0.020 | MAX Y | 0.018 | RMS X | 0.017 | SD R | 0.017 |
|  | 3 | RMS R | 0.015 | MAX Z | 0.014 | SE R | 0.013 | P25 X | 0.012 | MAX X | 0.011 |
|  | 4 | RMS Y | 0.021 | MAX R | 0.020 | SD X | 0.020 | MED Y | 0.018 | MED X | 0.016 |
| 11 | 1 | P75 X | 0.027 | P25 X | 0.027 | P75 R | 0.026 | RATIO Z | 0.026 | MAX Y | 0.025 |
|  | 2 | MAX X | 0.027 | RATIO Z | 0.026 | P25 X | 0.024 | SE R | 0.024 | MED X | 0.022 |
|  | 3 | SE X | 0.026 | MIN Z | 0.025 | MIN Y | 0.022 | P25 Z | 0.018 | MED R | 0.018 |
|  | 4 | P25 X | 0.025 | P75 R | 0.024 | MAX Y | 0.024 | RATIO Y | 0.024 | SE R | 0.023 |
| 12 | 1 | MED Z | 0.019 | MEAN R | 0.019 | RMS R | 0.018 | SE Y | 0.017 | P75 X | 0.017 |
|  | 2 | SE Y | 0.015 | RATIO Y | 0.014 | MEAN Z | 0.012 | P25 R | 0.012 | RATIO X | 0.011 |
|  | 3 | MIN Z | 0.027 | MEAN Z | 0.027 | RATIO Z | 0.026 | MED Y | 0.026 | MEAN Y | 0.025 |
|  | 4 | SD Z | 0.011 | MEAN Z | 0.009 | SE Z | 0.008 | P25 R | 0.008 | RMS R | 0.008 |
| 13 | 1 | RMS Z | 0.027 | MEAN Y | 0.026 | RMS R | 0.026 | RMS Y | 0.026 | P25 X | 0.026 |
|  | 2 | MEAN R | 0.023 | MEAN X | 0.021 | RATIO Y | 0.019 | RMS Z | 0.018 | MED X | 0.017 |
|  | 3 | SD X | 0.020 | P25 Z | 0.019 | MIN Z | 0.017 | MEAN X | 0.015 | SE Z | 0.015 |
|  | 4 | MEAN X | 0.028 | SD R | 0.026 | SE Z | 0.021 | MEAN Y | 0.017 | RMS X | 0.016 |
| 14 | 1 | RMS R | 0.028 | P75 R | 0.027 | RMS Z | 0.027 | SD R | 0.026 | SE Z | 0.025 |
|  | 2 | P25 X | 0.025 | P25 Z | 0.025 | P25 Y | 0.025 | MIN R | 0.025 | RATIO X | 0.024 |
|  | 3 | SE R | 0.024 | MED R | 0.023 | P25 Z | 0.017 | MIN R | 0.016 | SE X | 0.016 |
|  | 4 | MEAN Z | 0.015 | MAX Z | 0.015 | MED Z | 0.014 | SD Z | 0.012 | SE R | 0.012 |
| 15 | 1 | RMS Y | 0.023 | MEAN Y | 0.022 | RMS R | 0.022 | RMS X | 0.021 | MED X | 0.021 |
|  | 2 | MED Z | 0.032 | SE Y | 0.029 | MAX Y | 0.028 | SE X | 0.025 | SD Z | 0.022 |
|  | 3 | RMS X | 0.023 | SD X | 0.018 | RMS R | 0.017 | MAX Y | 0.017 | RATIO X | 0.016 |
|  | 4 | MIN Y | 0.019 | MED R | 0.019 | SE Z | 0.018 | SD R | 0.018 | MED X | 0.015 |
| 16 | 1 | RATIO Y | 0.027 | RMS Y | 0.024 | MED Y | 0.023 | P25 Z | 0.023 | P25 R | 0.020 |
|  | 2 | RATIO Y | 0.024 | RMS X | 0.023 | SE Z | 0.016 | SE X | 0.015 | MAX Z | 0.015 |
|  | 3 | MEAN Y | 0.027 | MIN Z | 0.026 | SE Z | 0.025 | SE R | 0.025 | RATIO Y | 0.024 |
|  | 4 | RATIO Y | 0.025 | P75X | 0.025 | MAX X | 0.023 | P25 Z | 0.023 | RMS Z | 0.022 |

| Partic. | Left-out Trial | Variable 21 | Variable 21 Imp. | Variable 22 | Variable 22 Imp. | Variable 23 | Variable 23 Imp. | Variable 24 | Variable 24 Imp. | Variable 25 | Variable 25 Imp. |
| --- | --- | --- | --- | --- | --- | --- | --- | --- | --- | --- | --- |
| 1 | 1 | RATIO Y | 0.023 | MED X | 0.023 | RMS Z | 0.023 | P75 Y | 0.023 | MIN Z | 0.022 |
|  | 2 | P25 Z | 0.024 | P75 X | 0.024 | P75 R | 0.023 | SD Z | 0.023 | SD Y | 0.022 |
|  | 3 | MEAN Y | 0.022 | MEAN R | 0.022 | P75 Z | 0.022 | P75 X | 0.021 | RATIO X | 0.021 |
|  | 4 | P75 Y | 0.019 | MIN R | 0.019 | MIN Y | 0.018 | MAX Y | 0.017 | P75 R | 0.017 |
| 2 | 1 | MAX X | 0.012 | MED Z | 0.011 | MIN Z | 0.011 | P25 Z | 0.009 | MEAN Y | 0.007 |
|  | 2 | MIN R | 0.017 | P25 Y | 0.016 | SE X | 0.016 | MIN Z | 0.015 | MEAN Y | 0.015 |
|  | 3 | P25 Y | 0.016 | P25 R | 0.016 | MIN X | 0.015 | SE X | 0.014 | P75 Y | 0.014 |
|  | 4 | MEAN Y | 0.006 | MAX X | 0.006 | MEAN Z | 0.005 | RMS X | 0.004 | SE R | 0.003 |
| 3 | 1 | P25 R | 0.024 | MIN R | 0.020 | MIN Z | 0.020 | MEAN R | 0.020 | RMS X | 0.019 |
|  | 2 | MED Z | 0.024 | MIN R | 0.024 | MAX R | 0.024 | RATIO X | 0.023 | RMS Y | 0.023 |
|  | 3 | MED Z | 0.014 | MIN X | 0.013 | MED R | 0.013 | MAX Z | 0.011 | MED X | 0.011 |
|  | 4 | P25 R | 0.015 | RMS R | 0.015 | RMS Y | 0.015 | MEAN Z | 0.014 | MAX Y | 0.014 |
| 4 | 1 | RATIO Y | 0.022 | SE Z | 0.022 | RATIO X | 0.022 | MAX Z | 0.021 | RATIO Z | 0.021 |
|  | 2 | MEAN R | 0.023 | P75 Z | 0.023 | MIN Z | 0.022 | RMS Y | 0.022 | SE Z | 0.022 |
|  | 3 | SE Z | 0.012 | SD X | 0.011 | SD Y | 0.011 | MEAN R | 0.010 | MAX R | 0.009 |
|  | 4 | SE Z | 0.012 | SD Y | 0.012 | MAX Z | 0.009 | RMS Y | 0.007 | SE Y | 0.007 |
| 5 | 1 | P25 Z | 0.015 | SD X | 0.014 | SD R | 0.014 | MEAN Z | 0.013 | RATIO Y | 0.013 |
|  | 2 | RATIO Y | 0.018 | SD X | 0.018 | RMS Y | 0.016 | MAX Z | 0.014 | RMS X | 0.013 |
|  | 3 | SD Z | 0.014 | RATIO X | 0.014 | SD X | 0.011 | MED Y | 0.011 | SE X | 0.010 |
|  | 4 | MEAN R | 0.016 | MAX Z | 0.015 | MED Z | 0.013 | P25 Y | 0.013 | SD X | 0.013 |
| 6 | 1 | STD Z | 0.015 | MAX Y | 0.014 | RMS R | 0.011 | MEAN Z | 0.011 | RATIO X | 0.010 |
|  | 2 | P75 X | 0.009 | SD X | 0.009 | MEAN Z | 0.009 | P75 Z | 0.009 | RMS Z | 0.008 |
|  | 3 | MAX R | 0.009 | MED Z | 0.009 | MED Y | 0.008 | MEAN Z | 0.008 | SD Z | 0.008 |
|  | 4 | SD Z | 0.024 | MED Y | 0.023 | SE Y | 0.022 | MEAN X | 0.022 | MEAN Z | 0.020 |
| 7 | 1 | P25 Z | 0.021 | RATIO X | 0.021 | MEAN Z | 0.020 | RMS X | 0.020 | MIN R | 0.020 |
|  | 2 | SE R | 0.020 | SE X | 0.020 | P75 Z | 0.019 | MEAN Z | 0.018 | P75 Y | 0.016 |
|  | 3 | MAX Z | 0.024 | MEAN X | 0.021 | P25 Z | 0.021 | RMS Z | 0.021 | MIN Z | 0.020 |
|  | 4 | P25 Z | 0.021 | MED Y | 0.021 | MEAN Z | 0.021 | P25 Y | 0.021 | P75 Z | 0.017 |
| 8 | 1 | SD X | 0.013 | RATIO X | 0.012 | RMS X | 0.012 | P25 Z | 0.011 | P75 R | 0.011 |
|  | 2 | P75 R | 0.014 | MIN R | 0.012 | P25 Z | 0.010 | MED X | 0.009 | SE Z | 0.008 |
|  | 3 | RATIO X | 0.025 | MEAN Y | 0.024 | SE Z | 0.024 | RMS Z | 0.024 | P25 X | 0.023 |
|  | 4 | SD Y | 0.015 | P25 Z | 0.015 | SD X | 0.012 | RMS X | 0.011 | MAX Y | 0.010 |
| 9 | 1 | P75 Y | 0.023 | SD R | 0.021 | RMS R | 0.020 | P25 Z | 0.020 | P25 R | 0.020 |
|  | 2 | MIN Z | 0.013 | P75 R | 0.010 | P25 Y | 0.009 | MEAN Z | 0.008 | MEAN R | 0.008 |
|  | 3 | MEAN R | 0.020 | SE Y | 0.019 | MEAN Y | 0.019 | P25 Z | 0.017 | MEAN Z | 0.016 |
|  | 4 | RMS R | 0.019 | MED X | 0.019 | P75 R | 0.019 | SD R | 0.018 | RMS Y | 0.014 |
| 10 | 1 | RATIO Y | 0.016 | P75 X | 0.016 | SD Z | 0.015 | MIN Y | 0.011 | MAX X | 0.008 |
|  | 2 | MED Z | 0.016 | MED R | 0.015 | MIN Y | 0.014 | SE X | 0.013 | P75 R | 0.012 |
|  | 3 | P25 Z | 0.008 | SD Z | 0.008 | SE Y | 0.008 | MAX Y | 0.007 | MEAN Y | 0.005 |
|  | 4 | SD R | 0.016 | RATIO Y | 0.016 | MIN Z | 0.016 | MIN X | 0.015 | SD Y | 0.015 |
| 11 | 1 | P25 Z | 0.025 | SE R | 0.024 | MIN Y | 0.024 | MED X | 0.024 | MEAN R | 0.023 |
|  | 2 | RATIO X | 0.022 | MED Y | 0.022 | MIN Y | 0.021 | MAX Y | 0.020 | MEAN Z | 0.020 |
|  | 3 | MAX R | 0.017 | SD Y | 0.016 | MED X | 0.014 | MAX X | 0.013 | RMS Y | 0.013 |
|  | 4 | P75 X | 0.023 | P75 Z | 0.022 | P75 Y | 0.022 | MED X | 0.021 | MEAN R | 0.021 |
| 12 | 1 | MIN X | 0.014 | P75 Y | 0.010 | RMS Z | 0.010 | MEAN Y | 0.009 | SD X | 0.009 |
|  | 2 | P75 Y | 0.011 | SD X | 0.009 | RMS X | 0.008 | P25 X | 0.008 | MEAN Y | 0.008 |
|  | 3 | MED X | 0.025 | MAX Y | 0.025 | MAX R | 0.024 | P25 X | 0.024 | P25 Z | 0.024 |
|  | 4 | SD X | 0.008 | P25 X | 0.007 | RMS Z | 0.007 | RATIO Z | 0.007 | RATIO Y | 0.006 |
| 13 | 1 | MAX Z | 0.025 | SE X | 0.025 | RMS X | 0.023 | P25 Z | 0.023 | MED Y | 0.022 |
|  | 2 | SD R | 0.017 | MIN Z | 0.017 | RATIO Z | 0.015 | MIN Y | 0.014 | P75 X | 0.014 |
|  | 3 | SD Z | 0.014 | MEAN Z | 0.014 | RATIO Y | 0.011 | MED R | 0.010 | RATIO Z | 0.008 |
|  | 4 | P25 Y | 0.015 | P75 Z | 0.015 | P25 Z | 0.014 | P25 R | 0.009 | MEAN Z | 0.007 |
| 14 | 1 | P75 Z | 0.025 | MEAN R | 0.025 | MED Z | 0.024 | MIN X | 0.024 | RMS X | 0.020 |
|  | 2 | SD X | 0.024 | SE R | 0.023 | P75 Z | 0.023 | RMS X | 0.021 | MEAN R | 0.020 |
|  | 3 | MIN Y | 0.015 | SD X | 0.015 | RATIO Z | 0.014 | MED Z | 0.013 | MEAN R | 0.012 |
|  | 4 | P75 Y | 0.012 | RMS Z | 0.010 | MAX R | 0.009 | MIN X | 0.008 | MIN Y | 0.007 |
| 15 | 1 | SE R | 0.020 | P25 Y | 0.017 | P75 Y | 0.016 | P75 Z | 0.015 | MEAN Z | 0.014 |
|  | 2 | MIN Y | 0.020 | RMS Y | 0.019 | MED X | 0.019 | SD Y | 0.016 | P25 Y | 0.014 |
|  | 3 | MAX X | 0.015 | P75 Z | 0.015 | MED X | 0.015 | SD Z | 0.015 | MED R | 0.015 |
|  | 4 | MAX Y | 0.015 | SE X | 0.013 | P25 Y | 0.013 | MIN X | 0.012 | P75 Y | 0.011 |
| 16 | 1 | SE Z | 0.019 | P75 Y | 0.017 | SE X | 0.017 | SE R | 0.017 | MAX Y | 0.016 |
|  | 2 | MAX X | 0.013 | MEAN R | 0.012 | P75 Y | 0.012 | MIN X | 0.008 | RMS Z | 0.007 |
|  | 3 | MEAN R | 0.024 | SD X | 0.023 | MIN X | 0.022 | SE X | 0.022 | RMS Z | 0.022 |
|  | 4 | RMS X | 0.022 | RATIO Z | 0.021 | MIN X | 0.021 | MAX Y | 0.021 | MIN Z | 0.020 |

| Partic. | Left-out Trial | Variable 26 | Variable 26 Imp. | Variable 27 | Variable 27 Imp. | Variable 28 | Variable 28 Imp. | Variable 29 | Variable 29 Imp. | Variable 30 | Variable 30 Imp. |
| --- | --- | --- | --- | --- | --- | --- | --- | --- | --- | --- | --- |
| 1 | 1 | MEAN R | 0.022 | MED Z | 0.022 | MAX Z | 0.021 | MED R | 0.021 | MIN X | 0.020 |
|  | 2 | MEAN R | 0.022 | P25 R | 0.022 | MIN X | 0.022 | SE Y | 0.019 | MAX Z | 0.019 |
|  | 3 | MIN Y | 0.020 | MEAN X | 0.020 | P25 X | 0.020 | RATIO Y | 0.020 | MIN X | 0.019 |
|  | 4 | MAX X | 0.017 | SE R | 0.016 | RMS Y | 0.016 | MAX R | 0.015 | P75 Z | 0.015 |
| 2 | 1 | P75 Y | 0.007 | P75 Z | 0.007 | SE R | 0.006 | RMS X | 0.005 | MED Y | 0.005 |
|  | 2 | SD R | 0.013 | P25 Z | 0.012 | RMS Z | 0.011 | RMS X | 0.011 | SD Z | 0.010 |
|  | 3 | P25 X | 0.014 | MAX X | 0.012 | RMS Z | 0.011 | RMS X | 0.010 | MED Z | 0.009 |
|  | 4 | P75 Y | 0.003 | MED Z | 0.003 | MIN X | 0.003 | P25 Z | 0.002 | SE X | 0.002 |
| 3 | 1 | RATIO X | 0.019 | P75 Z | 0.018 | RMS R | 0.018 | MAX R | 0.017 | MAX Y | 0.017 |
|  | 2 | RMS R | 0.022 | RMS Z | 0.022 | MAX Y | 0.021 | P75 Z | 0.020 | RATIO Y | 0.019 |
|  | 3 | RMS Y | 0.010 | SD Y | 0.010 | P25 R | 0.010 | SE Y | 0.009 | RATIO Y | 0.009 |
|  | 4 | SD Y | 0.014 | RATIO Y | 0.013 | MEAN R | 0.013 | MIN Z | 0.012 | MIN X | 0.012 |
| 4 | 1 | MEAN X | 0.021 | MIN R | 0.021 | RMS Z | 0.020 | SD X | 0.018 | MIN Y | 0.018 |
|  | 2 | MED Y | 0.022 | RMS R | 0.020 | P75 R | 0.020 | SE Y | 0.020 | P25 Y | 0.019 |
|  | 3 | MAX X | 0.008 | RMS X | 0.007 | P25 Y | 0.006 | MAX Z | 0.005 | MEAN X | 0.005 |
|  | 4 | P25 Y | 0.007 | SD X | 0.006 | RMS X | 0.006 | MEAN X | 0.005 | MIN R | 0.005 |
| 5 | 1 | MIN Z | 0.010 | MED Y | 0.010 | SE Z | 0.010 | P75 X | 0.007 | RMS X | 0.006 |
|  | 2 | P25 Z | 0.011 | MIN X | 0.011 | SE Y | 0.011 | P75 X | 0.009 | MEAN R | 0.009 |
|  | 3 | MIN Y | 0.008 | P75 X | 0.007 | P25 Y | 0.006 | RMS X | 0.005 | P25 Z | 0.004 |
|  | 4 | P25 Z | 0.011 | MEAN X | 0.011 | RMS X | 0.010 | SD R | 0.010 | SD Y | 0.008 |
| 6 | 1 | MEAN R | 0.009 | RMS X | 0.008 | P75 Z | 0.008 | MED Z | 0.008 | SD X | 0.008 |
|  | 2 | MEAN Y | 0.007 | RMS X | 0.006 | SD Z | 0.006 | SE Z | 0.006 | MED Y | 0.005 |
|  | 3 | RMS R | 0.008 | RATIO X | 0.008 | MED X | 0.008 | MEAN R | 0.007 | P75 R | 0.006 |
|  | 4 | SD X | 0.020 | P25 R | 0.019 | RMS Z | 0.019 | P75 Z | 0.019 | MIN Z | 0.018 |
| 7 | 1 | MAX Z | 0.019 | SE Z | 0.018 | P75 R | 0.017 | SE X | 0.016 | SE Y | 0.016 |
|  | 2 | MAX Z | 0.016 | MED Y | 0.015 | P25 Z | 0.015 | SD X | 0.013 | MEAN X | 0.011 |
|  | 3 | P75 Y | 0.020 | SE R | 0.019 | MAX R | 0.017 | RMS Y | 0.017 | P25 Y | 0.017 |
|  | 4 | SE R | 0.017 | RMS Y | 0.016 | SD Y | 0.016 | SE Z | 0.015 | P75 R | 0.015 |
| 8 | 1 | RMS Y | 0.010 | SD Y | 0.009 | RMS Z | 0.009 | MED Z | 0.008 | SD Z | 0.008 |
|  | 2 | MED Z | 0.008 | RMS X | 0.008 | MIN X | 0.008 | SD R | 0.007 | RMS R | 0.006 |
|  | 3 | SD Y | 0.023 | RMS X | 0.022 | MED R | 0.021 | P75 X | 0.021 | RMS Y | 0.021 |
|  | 4 | MED Z | 0.009 | RATIO Z | 0.009 | RMS Z | 0.008 | MAX X | 0.008 | MED R | 0.008 |
| 9 | 1 | MIN X | 0.019 | P75 R | 0.018 | P25 Y | 0.018 | RATIO Y | 0.017 | MEAN X | 0.017 |
|  | 2 | P25 Z | 0.007 | MED R | 0.007 | SE Y | 0.006 | RMS R | 0.005 | MIN X | 0.004 |
|  | 3 | P75 R | 0.014 | P75 Y | 0.014 | MAX Y | 0.014 | SD Y | 0.012 | MIN Z | 0.012 |
|  | 4 | MAX X | 0.014 | P25 R | 0.014 | SD Y | 0.011 | MED Y | 0.011 | P25 Z | 0.010 |
| 10 | 1 | SE X | 0.008 | SE Y | 0.008 | MEAN Y | 0.007 | SD R | 0.007 | RATIO X | 0.005 |
|  | 2 | RATIO X | 0.011 | MED Y | 0.011 | SE Y | 0.011 | MAX R | 0.011 | MAX X | 0.011 |
|  | 3 | SD R | 0.005 | MIN Y | 0.005 | MED Z | 0.005 | MAX R | 0.004 | RATIO X | 0.003 |
|  | 4 | MAX Y | 0.014 | MAX Z | 0.014 | RMS X | 0.013 | MEAN Y | 0.013 | MIN Y | 0.010 |
| 11 | 1 | MEAN Z | 0.021 | RMS Y | 0.021 | P25 R | 0.019 | P75 Y | 0.019 | RATIO Y | 0.018 |
|  | 2 | RATIO Y | 0.017 | MIN X | 0.017 | SE Y | 0.015 | MEAN R | 0.015 | MEAN X | 0.013 |
|  | 3 | SE R | 0.009 | SE Y | 0.009 | P75 Z | 0.007 | RATIO Y | 0.006 | MAX Y | 0.006 |
|  | 4 | MAX R | 0.021 | RATIO X | 0.020 | P25 Z | 0.020 | SE X | 0.019 | MIN R | 0.017 |
| 12 | 1 | SD R | 0.009 | SD Z | 0.009 | P25 X | 0.009 | RATIO X | 0.008 | RMS X | 0.008 |
|  | 2 | SD R | 0.008 | MAX R | 0.006 | RMS Z | 0.006 | MAX Y | 0.005 | P25 Z | 0.005 |
|  | 3 | MIN Y | 0.023 | RMS Y | 0.021 | SD X | 0.020 | P25 R | 0.020 | RMS Z | 0.020 |
|  | 4 | RMS X | 0.005 | P25 Y | 0.005 | MAX Y | 0.005 | RATIO X | 0.005 | P25 Z | 0.005 |
| 13 | 1 | MAX Y | 0.021 | P75 X | 0.021 | MED Z | 0.021 | MED X | 0.020 | P25 R | 0.019 |
|  | 2 | RATIO X | 0.012 | P75 X | 0.012 | MAX Y | 0.011 | SD Z | 0.010 | MEAN Z | 0.010 |
|  | 3 | MAX Z | 0.007 | P25 Y | 0.007 | P75 Z | 0.006 | MED Y | 0.006 | MED Z | 0.005 |
|  | 4 | MAX R | 0.007 | MED X | 0.005 | RATIO Y | 0.004 | MAX Y | 0.003 | P75 Y | 0.001 |
| 14 | 1 | MAX X | 0.019 | P25 Z | 0.018 | SE X | 0.018 | MAX R | 0.018 | P25 R | 0.017 |
|  | 2 | MIN X | 0.019 | P25 R | 0.019 | MEAN Y | 0.018 | MAX X | 0.018 | MEAN Z | 0.017 |
|  | 3 | SD Z | 0.012 | MAX Z | 0.012 | RMS X | 0.011 | MEAN Z | 0.010 | MAX X | 0.009 |
|  | 4 | SE X | 0.007 | MAX X | 0.006 | MEAN R | 0.006 | P25 Z | 0.006 | MED Y | 0.005 |
| 15 | 1 | P75 X | 0.014 | SD Z | 0.013 | SE X | 0.013 | MEAN X | 0.011 | MIN X | 0.011 |
|  | 2 | MEAN R | 0.013 | RATIO X | 0.012 | P75 R | 0.009 | RATIO Z | 0.009 | MED Y | 0.009 |
|  | 3 | MIN Y | 0.013 | MAX Z | 0.013 | SE R | 0.013 | P25 Y | 0.011 | RMS Z | 0.010 |
|  | 4 | SD Z | 0.011 | MAX Z | 0.011 | P75 Z | 0.010 | SE R | 0.010 | P75 R | 0.009 |
| 16 | 1 | P75 Z | 0.014 | MIN Z | 0.013 | RMS Z | 0.012 | MIN Y | 0.012 | MAX R | 0.012 |
|  | 2 | MED Y | 0.007 | MIN Y | 0.007 | P75 R | 0.006 | MAX Y | 0.006 | RATIO X | 0.006 |
|  | 3 | P75 Y | 0.022 | RATIO Z | 0.020 | P25 R | 0.019 | MAX X | 0.019 | P75 Z | 0.018 |
|  | 4 | SE R | 0.020 | SE Z | 0.018 | SD Z | 0.018 | P75 Z | 0.017 | RATIO X | 0.017 |

| Partic. | Left-out Trial | Variable 31 | Variable 31 Imp. | Variable 32 | Variable 32 Imp. | Variable 33 | Variable 33 Imp. | Variable 34 | Variable 34 Imp. | Variable 35 | Variable 35 Imp. |
| --- | --- | --- | --- | --- | --- | --- | --- | --- | --- | --- | --- |
| 1 | 1 | SE Z | 0.020 | SD Y | 0.020 | MAX X | 0.019 | P75 X | 0.019 | SE R | 0.016 |
|  | 2 | RMS R | 0.019 | RMS Y | 0.018 | SD X | 0.017 | RATIO Y | 0.017 | RMS X | 0.017 |
|  | 3 | P75 Y | 0.016 | MED R | 0.016 | P75 R | 0.014 | P25 Z | 0.013 | SD X | 0.011 |
|  | 4 | P25 Z | 0.015 | MEAN R | 0.015 | SD Y | 0.014 | RATIO Y | 0.014 | P25 R | 0.013 |
| 2 | 1 | SD X | 0.004 | SE X | 0.004 | SD Z | 0.004 | RMS Z | 0.003 | MAX Z | 0.003 |
|  | 2 | MEAN X | 0.010 | MAX Z | 0.009 | MIN X | 0.007 | RATIO Z | 0.006 | MED Z | 0.005 |
|  | 3 | P75 Z | 0.008 | P25 Z | 0.007 | MAX Z | 0.007 | MEAN X | 0.007 | RATIO Z | 0.006 |
|  | 4 | SD Z | 0.002 | RMS Z | 0.001 | P75 Z | 0.001 | MAX Z | 0.001 | RATIO Z | 0.001 |
| 3 | 1 | SE R | 0.017 | MEAN Z | 0.015 | RMS Y | 0.013 | SE Y | 0.013 | SD Y | 0.012 |
|  | 2 | SD Y | 0.017 | MEAN R | 0.017 | SE Y | 0.017 | MIN Z | 0.017 | SD R | 0.015 |
|  | 3 | P25 Y | 0.009 | P75 Y | 0.007 | SD R | 0.006 | P75 Z | 0.006 | MAX R | 0.004 |
|  | 4 | SE Y | 0.009 | SD R | 0.009 | P75 Z | 0.006 | MAX X | 0.005 | MAX R | 0.004 |
| 4 | 1 | MAX Y | 0.017 | MAX X | 0.017 | MED Y | 0.016 | RMS X | 0.016 | MAX R | 0.016 |
|  | 2 | MEAN Y | 0.019 | SD R | 0.018 | P25 R | 0.017 | RATIO Z | 0.016 | MAX Y | 0.015 |
|  | 3 | MIN X | 0.005 | SE Y | 0.004 | MED Y | 0.004 | MED X | 0.004 | P25 R | 0.004 |
|  | 4 | MAX Y | 0.004 | MIN Z | 0.004 | MIN Y | 0.004 | MEAN R | 0.003 | RMS R | 0.003 |
| 5 | 1 | P25 R | 0.006 | MEAN R | 0.006 | RMS R | 0.004 | MIN X | 0.004 | P25 Y | 0.002 |
|  | 2 | SD R | 0.009 | MEAN Y | 0.008 | MIN Y | 0.008 | P75 Z | 0.007 | MEAN X | 0.007 |
|  | 3 | MIN X | 0.004 | MEAN Y | 0.003 | SD R | 0.003 | P75 Z | 0.003 | MEAN X | 0.001 |
|  | 4 | MIN Y | 0.008 | RMS Y | 0.008 | MEAN Y | 0.007 | P75 Z | 0.007 | P75 X | 0.007 |
| 6 | 1 | MIN Z | 0.007 | SE R | 0.007 | SE Y | 0.007 | MED Y | 0.006 | P75 R | 0.005 |
|  | 2 | MIN R | 0.005 | SE R | 0.005 | MEAN X | 0.004 | MIN Z | 0.003 | MIN Y | 0.003 |
|  | 3 | MEAN X | 0.006 | SD X | 0.005 | SE R | 0.004 | MAX Y | 0.003 | MIN R | 0.003 |
|  | 4 | MEAN R | 0.018 | RMS X | 0.017 | MAX Y | 0.016 | MIN Y | 0.015 | MIN X | 0.015 |
| 7 | 1 | SD R | 0.016 | MEAN Y | 0.015 | SE R | 0.015 | SD Y | 0.014 | MEAN X | 0.016 |
|  | 2 | SD Y | 0.009 | P75 R | 0.008 | MAX Y | 0.008 | RMS Y | 0.007 | SD R | 0.007 |
|  | 3 | MED Y | 0.016 | MAX Y | 0.016 | SE Z | 0.016 | RATIO Y | 0.015 | SD Y | 0.014 |
|  | 4 | SE X | 0.014 | P75 X | 0.014 | SD R | 0.014 | RATIO Y | 0.014 | MAX Y | 0.013 |
| 8 | 1 | MIN Y | 0.008 | MAX X | 0.007 | RMS R | 0.007 | RATIO Y | 0.007 | SD R | 0.006 |
|  | 2 | SD X | 0.006 | MAX Y | 0.005 | MAX R | 0.005 | P75 X | 0.004 | RATIO Z | 0.004 |
|  | 3 | SD X | 0.019 | MED Z | 0.019 | MIN X | 0.018 | MAX X | 0.018 | MAX Y | 0.017 |
|  | 4 | RATIO X | 0.008 | MIN X | 0.007 | P75 X | 0.007 | SE Z | 0.007 | SD R | 0.006 |
| 9 | 1 | SD Y | 0.017 | MED R | 0.017 | MEAN R | 0.016 | RMS Y | 0.016 | MIN Y | 0.015 |
|  | 2 | MAX Z | 0.004 | SD R | 0.004 | MED Y | 0.003 | RMS Y | 0.003 | RATIO Y | 0.003 |
|  | 3 | MEAN X | 0.011 | P25 Y | 0.011 | MED X | 0.010 | MIN X | 0.010 | RMS Y | 0.009 |
|  | 4 | P25 Y | 0.010 | MAX Z | 0.009 | MIN X | 0.009 | RATIO Y | 0.008 | MIN Y | 0.007 |
| 10 | 1 | P75 Z | 0.005 | MAX R | 0.005 | MAX Y | 0.005 | P25 X | 0.005 | MAX Z | 0.005 |
|  | 2 | MEAN Y | 0.010 | MAX Z | 0.009 | MIN Z | 0.009 | P25 X | 0.009 | P75 X | 0.009 |
|  | 3 | MEAN Z | 0.003 | MED X | 0.003 | P75 X | 0.001 | P75 Z | 0.001 | MED Y | 0.000 |
|  | 4 | P75 X | 0.009 | P75 Y | 0.009 | SD Z | 0.008 | P25 X | 0.007 | SE Y | 0.007 |
| 11 | 1 | SE Y | 0.018 | MED R | 0.017 | P75 Z | 0.015 | MEAN Y | 0.015 | MIN X | 0.015 |
|  | 2 | P75 Y | 0.013 | P75 Z | 0.013 | RMS Y | 0.013 | SD Y | 0.013 | MED Z | 0.012 |
|  | 3 | MEAN R | 0.005 | MAX Z | 0.005 | MEAN Y | 0.004 | MIN X | 0.004 | MEAN X | 0.004 |
|  | 4 | MED R | 0.016 | SE Y | 0.016 | RMS Y | 0.015 | P25 R | 0.015 | MEAN Y | 0.015 |
| 12 | 1 | MEAN Z | 0.007 | RATIO Z | 0.006 | P25 Z | 0.004 | P25 Y | 0.003 | P75 Z | 0.002 |
|  | 2 | P25 Y | 0.005 | SD Z | 0.005 | SE Z | 0.004 | MIN Z | 0.004 | MIN X | 0.003 |
|  | 3 | SE Y | 0.017 | P75 Y | 0.016 | RMS X | 0.015 | MED Z | 0.013 | SD R | 0.012 |
|  | 4 | P75 Y | 0.005 | MIN R | 0.004 | MAX R | 0.004 | SD R | 0.004 | SE Y | 0.002 |
| 13 | 1 | MEAN R | 0.019 | P75 Z | 0.018 | MIN X | 0.018 | P75 Y | 0.016 | RATIO Y | 0.016 |
|  | 2 | MED R | 0.010 | MIN X | 0.009 | P75 R | 0.008 | P25 R | 0.008 | MIN R | 0.007 |
|  | 3 | MAX Y | 0.005 | MIN X | 0.004 | MEAN Y | 0.003 | P75 R | 0.003 | P75 Y | 0.002 |
|  | 4 | SE R | 0.000 | MIN X | 0.000 | MED Y | 0.000 | MIN R | 0.000 | P75 R | 0.000 |
| 14 | 1 | MEAN Z | 0.017 | MED Y | 0.015 | P75 Y | 0.015 | MIN Y | 0.015 | MAX Z | 0.014 |
|  | 2 | SE X | 0.017 | P75 Y | 0.016 | MIN Z | 0.015 | MIN Y | 0.014 | MED R | 0.013 |
|  | 3 | RMS Z | 0.009 | MAX R | 0.008 | P75 Y | 0.007 | MED Y | 0.006 | MIN Z | 0.006 |
|  | 4 | MEAN X | 0.005 | P25 Y | 0.005 | SD R | 0.004 | P25 R | 0.004 | MIN Z | 0.004 |
| 15 | 1 | MED R | 0.010 | MIN Y | 0.009 | MIN R | 0.008 | RATIO Z | 0.006 | MED Y | 0.006 |
|  | 2 | MEAN Y | 0.007 | P75 X | 0.007 | P75 Z | 0.006 | MIN R | 0.005 | MIN X | 0.005 |
|  | 3 | SE X | 0.009 | MIN X | 0.008 | SD R | 0.008 | MIN R | 0.007 | P75 R | 0.007 |
|  | 4 | MED Y | 0.009 | P25 Z | 0.009 | MEAN X | 0.008 | MED Z | 0.008 | MIN Z | 0.008 |
| 16 | 1 | MAX Z | 0.011 | MED X | 0.008 | MEAN R | 0.008 | MIN X | 0.005 | RATIO X | 0.005 |
|  | 2 | MIN R | 0.005 | P75 X | 0.005 | P25 R | 0.005 | P75 Z | 0.005 | MED R | 0.004 |
|  | 3 | RATIO X | 0.018 | MAX Y | 0.017 | MEAN X | 0.016 | MED Y | 0.015 | MED X | 0.014 |
|  | 4 | MIN R | 0.016 | RMS Y | 0.016 | MIN Y | 0.016 | P25 Y | 0.015 | MEAN X | 0.015 |

| Partic. | Left-out Trial | Variable 36 | Variable 36 Imp. | Variable 37 | Variable 37 Imp. | Variable 38 | Variable 38 Imp. | Variable 39 | Variable 39 Imp. |
| --- | --- | --- | --- | --- | --- | --- | --- | --- | --- |
| 1 | 1 | MIN Y | 0.016 | SD X | 0.014 | RMS X | 0.013 | P75 R | 0.012 |
|  | 2 | P75 Y | 0.016 | SE R | 0.015 | SE Z | 0.015 | MED R | 0.014 |
|  | 3 | MED Y | 0.009 | P25 R | 0.008 | MED X | 0.008 | P25 Y | 0.006 |
|  | 4 | SD X | 0.011 | SE Z | 0.011 | RMS X | 0.009 | MIN X | 0.006 |
| 2 | 1 | SE Z | 0.003 | MIN X | 0.002 | MEAN X | 0.002 | RATIO Z | 0.002 |
|  | 2 | SE Z | 0.005 | MED Y | 0.005 | P75 Y | 0.003 | P75 Z | 0.003 |
|  | 3 | MED Y | 0.006 | SD R | 0.006 | SE Z | 0.003 | MIN R | 0.002 |
|  | 4 | MED Y | 0.001 | SD R | 0.001 | MEAN X | 0.000 | SE Z | 0.000 |
| 3 | 1 | RATIO Y | 0.010 | MEAN Y | 0.008 | MEAN X | 0.008 | MIN Y | 0.005 |
|  | 2 | MEAN Z | 0.013 | P25 X | 0.012 | MIN Y | 0.010 | MEAN X | 0.009 |
|  | 3 | P25 X | 0.004 | MIN Y | 0.003 | MEAN X | 0.003 | MIN R | 0.002 |
|  | 4 | MEAN X | 0.004 | MIN Y | 0.003 | P25 X | 0.003 | MIN R | 0.003 |
| 4 | 1 | MIN X | 0.015 | SD R | 0.012 | MIN Z | 0.011 | P25 Y | 0.010 |
|  | 2 | MIN X | 0.013 | MED X | 0.012 | MEAN X | 0.011 | MAX Z | 0.006 |
|  | 3 | SD R | 0.003 | MIN Z | 0.003 | MIN R | 0.003 | MAX Y | 0.002 |
|  | 4 | P75 R | 0.002 | P25 R | 0.001 | MED X | 0.000 | MIN X | 0.000 |
| 5 | 1 | MEAN X | 0.002 | MIN R | 0.001 | MEAN Y | 0.000 | P75 Z | 0.000 |
|  | 2 | RMS R | 0.007 | P25 Y | 0.007 | P25 R | 0.007 | MIN R | 0.005 |
|  | 3 | MEAN R | 0.001 | P25 R | 0.000 | MIN R | 0.000 | RMS R | 0.000 |
|  | 4 | RMS R | 0.006 | P25 R | 0.006 | RATIO Y | 0.006 | MIN R | 0.004 |
| 6 | 1 | MEAN X | 0.005 | MIN Y | 0.004 | MIN R | 0.003 | MIN X | 0.001 |
|  | 2 | P75 R | 0.003 | MEAN R | 0.002 | MIN X | 0.002 | MAX Z | 0.002 |
|  | 3 | MIN X | 0.002 | MIN Y | 0.002 | MIN Z | 0.001 | RMS X | 0.000 |
|  | 4 | P75 R | 0.015 | MIN R | 0.015 | RATIO X | 0.014 | SE R | 0.013 |
| 7 | 1 | RATIO Y | 0.013 | RMS Y | 0.013 | MIN Y | 0.011 | MAX Y | 0.010 |
|  | 2 | RATIO Y | 0.006 | MED Z | 0.006 | SE Y | 0.005 | SE Z | 0.004 |
|  | 3 | MIN Y | 0.013 | P75 R | 0.012 | SE Y | 0.011 | SD R | 0.010 |
|  | 4 | MAX Z | 0.013 | MEAN X | 0.012 | MIN Y | 0.010 | SE Y | 0.009 |
| 8 | 1 | MIN R | 0.006 | MED R | 0.006 | MIN X | 0.006 | MAX R | 0.004 |
|  | 2 | P75 Z | 0.003 | MAX X | 0.003 | RMS Z | 0.002 | SD Z | 0.001 |
|  | 3 | MIN R | 0.017 | P75 Z | 0.017 | MAX R | 0.016 | RMS R | 0.014 |
|  | 4 | MIN R | 0.006 | SD Z | 0.006 | MIN Y | 0.005 | RMS R | 0.004 |
| 9 | 1 | SE Y | 0.015 | MED Y | 0.015 | MAX Z | 0.014 | MIN R | 0.011 |
|  | 2 | MEAN X | 0.002 | SD Y | 0.002 | MIN R | 0.002 | MIN Y | 0.001 |
|  | 3 | MIN Y | 0.007 | RATIO Y | 0.007 | MAX Z | 0.006 | MIN R | 0.003 |
|  | 4 | MED R | 0.007 | MEAN R | 0.007 | MEAN X | 0.007 | MIN R | 0.006 |
| 10 | 1 | MIN Z | 0.004 | MED X | 0.004 | MEAN X | 0.003 | MIN R | 0.003 |
|  | 2 | MED X | 0.006 | MEAN X | 0.006 | P75 Z | 0.006 | MIN R | 0.002 |
|  | 3 | MIN Z | 0.000 | MIN R | 0.000 | SE X | 0.000 | MEAN X | 0.000 |
|  | 4 | RATIO X | 0.005 | MIN R | 0.004 | MEAN X | 0.004 | P75 Z | 0.003 |
| 11 | 1 | MIN R | 0.014 | MAX Z | 0.013 | MED Z | 0.013 | MEAN X | 0.012 |
|  | 2 | MAX Z | 0.012 | P25 R | 0.011 | MIN R | 0.011 | MEAN Y | 0.010 |
|  | 3 | MED Z | 0.004 | P25 R | 0.004 | MIN R | 0.003 | P75 Y | 0.003 |
|  | 4 | MIN X | 0.013 | MEAN X | 0.013 | MED Z | 0.012 | MAX Z | 0.011 |
| 12 | 1 | MIN R | 0.002 | P25 R | 0.001 | MIN Z | 0.001 | MAX Z | 0.000 |
|  | 2 | RATIO Z | 0.003 | MIN R | 0.003 | P75 Z | 0.001 | MAX Z | 0.001 |
|  | 3 | P75 Z | 0.011 | P25 Y | 0.010 | MIN X | 0.010 | MAX Z | 0.008 |
|  | 4 | MIN X | 0.002 | P75 Z | 0.001 | MIN Z | 0.001 | MAX Z | 0.001 |
| 13 | 1 | P75 R | 0.016 | MED R | 0.015 | P25 Y | 0.013 | MIN R | 0.012 |
|  | 2 | MED Z | 0.006 | MED Y | 0.005 | P25 Y | 0.005 | P75 Y | 0.004 |
|  | 3 | MED X | 0.002 | MIN R | 0.001 | P75 X | 0.001 | P25 R | 0.001 |
|  | 4 | MAX Z | 0.000 | MED Z | 0.000 | P75 X | 0.000 | MED R | 0.000 |
| 14 | 1 | MIN Z | 0.012 | P25 Y | 0.012 | MEAN X | 0.011 | MAX Y | 0.008 |
|  | 2 | MAX Y | 0.011 | MED Y | 0.010 | MAX Z | 0.010 | MEAN X | 0.009 |
|  | 3 | P25 Y | 0.005 | MEAN X | 0.005 | MIN X | 0.004 | MAX Y | 0.003 |
|  | 4 | SD X | 0.003 | MAX Y | 0.003 | RMS X | 0.003 | RMS R | 0.003 |
| 15 | 1 | RATIO X | 0.006 | MAX Y | 0.005 | MAX Z | 0.002 | P75 R | 0.002 |
|  | 2 | MAX Z | 0.004 | P75 Y | 0.003 | MED R | 0.002 | MEAN X | 0.002 |
|  | 3 | MEAN X | 0.005 | RATIO Z | 0.004 | P75 Y | 0.004 | MED Y | 0.004 |
|  | 4 | RATIO Z | 0.007 | RMS Z | 0.006 | MIN R | 0.005 | RATIO X | 0.004 |
| 16 | 1 | MIN R | 0.004 | MEAN X | 0.004 | P25 Y | 0.003 | MED R | 0.003 |
|  | 2 | RATIO Z | 0.004 | P25 Y | 0.003 | MEAN X | 0.003 | MED X | 0.002 |
|  | 3 | P25 Y | 0.013 | MIN Y | 0.013 | MIN R | 0.012 | MED R | 0.011 |
|  | 4 | MED Y | 0.014 | MEAN Y | 0.014 | MED R | 0.013 | MED X | 0.013 |

*Supplementary Table 7. Variable importance rankings and values from the subject-specific random forest classifiers for Experiment 1. MAX = maximum, MEAN = mean, MED = median, MIN = minimum, P25 = 25^th^ percentile, P75 = 75^th^ percentile, RATIO = ratio of single-axis RMS to RMS of the resultant axis, RMS = root mean square, SE = sample entropy, SD = standard deviation, R = resultant axis, X = vertical axis, Y = medio-lateral axis, Z = anterior-posterior axis*
