# Supplementary material for: Use of subject-specific models to detect fatigue-related changes in running biomechanics: a random forest approach
Source: Front Sports Act Living. 2023 Dec 21;5:1283316. doi: 10.3389/fspor.2023.1283316 (PMC10768007; doi:10.3389/fspor.2023.1283316)
Supplement: Supplementary file 8 [file Table8.docx]

| Left-out Participant | Variable 1 | Variable 1 Imp. | Variable 2 | Variable 2 Imp. | Variable 3 | Variable 3 Imp. | Variable 4 | Variable 4 Imp. | Variable 5 | Variable 5 Imp. |
| --- | --- | --- | --- | --- | --- | --- | --- | --- | --- | --- |
| 1 | MED X | 0.072 | MAX X | 0.060 | SE X | 0.059 | SD Y | 0.050 | SE Z | 0.048 |
| 2 | MED X | 0.052 | MAX X | 0.050 | SD Y | 0.049 | RMS Y | 0.041 | SE Z | 0.038 |
| 3 | MAX X | 0.050 | MED X | 0.047 | SE X | 0.045 | RMS Y | 0.043 | SD Y | 0.041 |
| 4 | MAX X | 0.061 | MED X | 0.051 | RATIO Y | 0.044 | RMS Y | 0.042 | RATIO X | 0.041 |
| 5 | MAX X | 0.090 | RMS Y | 0.058 | SD Y | 0.058 | SE Z | 0.055 | SE X | 0.052 |
| 6 | MAX X | 0.078 | MED X | 0.060 | SE X | 0.053 | SE Z | 0.049 | SD Y | 0.040 |
| 7 | MAX X | 0.069 | RMS Y | 0.060 | SD Y | 0.052 | MED X | 0.049 | RATIO X | 0.042 |
| 8 | SE Z | 0.042 | SE X | 0.041 | MED X | 0.040 | P25 X | 0.037 | MAX X | 0.037 |
| 9 | MED X | 0.041 | SE Z | 0.038 | SE X | 0.036 | P25 X | 0.033 | SE R | 0.032 |
| 10 | MAX X | 0.064 | RMS Z | 0.057 | MED X | 0.056 | SSD Z | 0.051 | RMS R | 0.049 |
| 11 | MAX X | 0.090 | MED X | 0.057 | SD Y | 0.056 | SE Z | 0.053 | RMS R | 0.052 |
| 12 | SE Z | 0.048 | MED X | 0.046 | P25 X | 0.044 | RMS Y | 0.038 | RATIO Y | 0.038 |
| 13 | MAX X | 0.100 | MED X | 0.097 | SD Y | 0.055 | RMS Y | 0.054 | RATIO Y | 0.053 |
| 14 | MED X | 0.045 | SE Z | 0.041 | MAX X | 0.040 | SE X | 0.038 | P25 X | 0.037 |
| 15 | SD Y | 0.107 | P25 X | 0.090 | RMS Y | 0.084 | MAX X | 0.084 | RATIO Y | 0.064 |
| 16 | MED X | 0.071 | MAX X | 0.067 | RMS Y | 0.047 | SE Z | 0.046 | SE X | 0.046 |

| Left-out Participant | Variable 6 | Variable 6 Imp. | Variable 7 | Variable 7 Imp. | Variable 8 | Variable 8 Imp. | Variable 9 | Variable 9 Imp. | Variable 10 | Variable 10 Imp. |
| --- | --- | --- | --- | --- | --- | --- | --- | --- | --- | --- |
| 1 | P25 X | 0.042 | RMS Y | 0.039 | P75 R | 0.033 | RATIO Y | 0.033 | SE Y | 0.030 |
| 2 | SE X | 0.038 | SE Y | 0.037 | P75 R | 0.035 | RATIO Y | 0.032 | RMS Z | 0.031 |
| 3 | RMS R | 0.039 | P25 X | 0.038 | SE Z | 0.037 | RMS Z | 0.036 | RATIO X | 0.033 |
| 4 | RMS Z | 0.037 | SD Y | 0.035 | SE Y | 0.034 | SE Z | 0.033 | RATIO Z | 0.033 |
| 5 | MED X | 0.045 | RATIO Y | 0.044 | RMS R | 0.039 | RATIO X | 0.035 | P25 X | 0.035 |
| 6 | RMS Y | 0.036 | P25 X | 0.035 | RMS Z | 0.035 | RMS R | 0.034 | RATIO Y | 0.033 |
| 7 | RMS R | 0.041 | SE X | 0.037 | RATIO Y | 0.036 | P75 R | 0.035 | SE Z | 0.035 |
| 8 | SD Y | 0.035 | RMS Z | 0.031 | RMS Y | 0.031 | SD Z | 0.030 | SE R | 0.030 |
| 9 | SD Y | 0.032 | MAX X | 0.031 | RMS Y | 0.031 | RATIO Y | 0.030 | P75 X | 0.030 |
| 10 | MEAN R | 0.045 | P25 R | 0.041 | MAX R | 0.038 | P25 X | 0.037 | RATIO Z | 0.036 |
| 11 | SE X | 0.052 | P25 X | 0.046 | RMS Y | 0.045 | MEAN R | 0.035 | RATIO Y | 0.033 |
| 12 | SD Y | 0.037 | SD Z | 0.034 | SE X | 0.032 | SD Y | 0.031 | P75 X | 0.030 |
| 13 | P75 R | 0.051 | SE X | 0.049 | SE Z | 0.048 | RMS R | 0.036 | RATIO X | 0.032 |
| 14 | RMS Z | 0.031 | SD Y | 0.031 | SD Z | 0.030 | RATIO Y | 0.030 | MEAN Z | 0.029 |
| 15 | SE Y | 0.060 | MEAN R | 0.051 | RMS R | 0.049 | MAX Y | 0.047 | MED X | 0.044 |
| 16 | SD Y | 0.046 | P25 X | 0.043 | RATIO Y | 0.035 | MEAN R | 0.031 | SD Z | 0.029 |

| Left-out Participant | Variable 11 | Variable 11 Imp. | Variable 12 | Variable 12 Imp. | Variable 13 | Variable 13 Imp. | Variable 14 | Variable 14 Imp. | Variable 15 | Variable 15 Imp. |
| --- | --- | --- | --- | --- | --- | --- | --- | --- | --- | --- |
| 1 | RATIO X | 0.030 | RMS R | 0.028 | MEAN R | 0.028 | MED R | 0.026 | RMS Z | 0.026 |
| 2 | SD Z | 0.030 | RMS R | 0.029 | MEAN R | 0.029 | RATIO X | 0.029 | P25 X | 0.028 |
| 3 | SE Y | 0.032 | MEAN R | 0.031 | SD Z | 0.031 | RATIO Y | 0.029 | MIN Z | 0.028 |
| 4 | P75 R | 0.032 | SD Z | 0.032 | MEAN R | 0.031 | P25 R | 0.031 | SE X | 0.030 |
| 5 | P75 R | 0.035 | MED R | 0.030 | MEAN R | 0.028 | SD Z | 0.027 | RMS Z | 0.026 |
| 6 | MEAN R | 0.032 | RATIO X | 0.030 | SE Y | 0.027 | SD Z | 0.027 | P75 R | 0.026 |
| 7 | SE Y | 0.034 | MEAN R | 0.034 | RMS Z | 0.030 | MAX R | 0.029 | MAX Y | 0.028 |
| 8 | P75 X | 0.029 | RATIO Z | 0.028 | MEAN Z | 0.027 | RATIO Y | 0.027 | SD R | 0.027 |
| 9 | SD Z | 0.030 | RMS R | 0.030 | SE Y | 0.029 | MEAN Z | 0.029 | RMS Z | 0.027 |
| 10 | SD Y | 0.036 | MIN Z | 0.032 | RATIO Y | 0.031 | SE Y | 0.030 | P75 R | 0.027 |
| 11 | MED R | 0.030 | RMS Z | 0.028 | SE Y | 0.027 | MAX R | 0.027 | P75 Z | 0.025 |
| 12 | MEAN Z | 0.028 | RMS Z | 0.028 | SE R | 0.028 | SD R | 0.028 | P25 R | 0.027 |
| 13 | MEAN R | 0.027 | MED R | 0.026 | SE Y | 0.026 | RMS Z | 0.022 | MAX R | 0.022 |
| 14 | RMS Y | 0.029 | RATIO Z | 0.029 | SE R | 0.028 | MEAN R | 0.028 | RMS R | 0.028 |
| 15 | MAX R | 0.036 | RATIO X | 0.033 | P75 R | 0.024 | MIN Z | 0.023 | P75 Z | 0.020 |
| 16 | RMS Z | 0.028 | RATIO X | 0.028 | P75 R | 0.028 | MED R | 0.028 | SE Y | 0.027 |

| Left-out Participant | Variable 16 | Variable 16 Imp. | Variable 17 | Variable 17 Imp. | Variable 18 | Variable 18 Imp. | Variable 19 | Variable 19 Imp. | Variable 20 | Variable 20 Imp. |
| --- | --- | --- | --- | --- | --- | --- | --- | --- | --- | --- |
| 1 | SD Z | 0.025 | P25 R | 0.025 | SE R | 0.024 | P75 X | 0.022 | RATIO Z | 0.022 |
| 2 | RATIO Z | 0.027 | MED R | 0.025 | MAX Y | 0.025 | MIN Z | 0.025 | MEAN Z | 0.024 |
| 3 | MED R | 0.026 | P75 R | 0.026 | P75 Y | 0.025 | RATIO Z | 0.024 | SD R | 0.024 |
| 4 | RMS R | 0.027 | SD R | 0.025 | P25 X | 0.024 | MAX Y | 0.024 | MED R | 0.023 |
| 5 | SE Y | 0.026 | MIN Z | 0.024 | MAX Y | 0.023 | MAX R | 0.022 | MIN Y | 0.020 |
| 6 | MIN Z | 0.026 | SD R | 0.025 | MED R | 0.023 | RATIO Z | 0.023 | SE R | 0.023 |
| 7 | SD Z | 0.028 | MED R | 0.028 | MIN Z | 0.022 | P25 X | 0.022 | SD R | 0.021 |
| 8 | RATIO X | 0.027 | P25 Z | 0.026 | P75 R | 0.026 | SE Y | 0.026 | SD X | 0.025 |
| 9 | P75 R | 0.027 | P25 R | 0.027 | MEAN R | 0.027 | SD R | 0.026 | P25 Z | 0.026 |
| 10 | RATIO X | 0.026 | RMS Y | 0.026 | SE Z | 0.025 | SE X | 0.025 | MIN Y | 0.022 |
| 11 | SD R | 0.024 | MIN Z | 0.022 | RATIO X | 0.022 | P25 R | 0.022 | MIN Y | 0.021 |
| 12 | P75 R | 0.027 | RATIO Z | 0.027 | P25 Z | 0.026 | MAX X | 0.025 | P25 Y | 0.025 |
| 13 | MEAN Z | 0.022 | SD R | 0.020 | MAX Y | 0.019 | MIN Z | 0.019 | P25 X | 0.018 |
| 14 | SE Y | 0.027 | SD R | 0.026 | P25 Z | 0.025 | RATIO X | 0.025 | P25 R | 0.024 |
| 15 | SE X | 0.019 | SD Z | 0.016 | P25 R | 0.016 | MIN Y | 0.015 | MIN X | 0.014 |
| 16 | RMS R | 0.026 | RATIO Z | 0.025 | SD R | 0.024 | MIN Z | 0.024 | P75 Y | 0.023 |

| Left-out Participant | Variable 21 | Variable 21 Imp. | Variable 22 | Variable 22 Imp. | Variable 23 | Variable 23 Imp. | Variable 24 | Variable 24 Imp. | Variable 25 | Variable 25 Imp. |
| --- | --- | --- | --- | --- | --- | --- | --- | --- | --- | --- |
| 1 | SD X | 0.021 | MIN Z | 0.020 | MAX Y | 0.019 | P75 Y | 0.018 | MEAN Z | 0.018 |
| 2 | SD R | 0.023 | MAX R | 0.022 | P25 Z | 0.021 | SE R | 0.021 | P25 R | 0.021 |
| 3 | MAX Y | 0.023 | SD X | 0.023 | MAX R | 0.022 | MEAN Z | 0.022 | SE R | 0.022 |
| 4 | MAX R | 0.022 | MIN Y | 0.022 | MIN Z | 0.022 | P25 Y | 0.021 | SE R | 0.021 |
| 5 | RATIO Z | 0.019 | SD R | 0.018 | P75 Z | 0.018 | P75 X | 0.017 | P25 R | 0.017 |
| 6 | P75 X | 0.022 | P75 Z | 0.021 | P25 R | 0.021 | MEAN Z | 0.021 | MAX Y | 0.020 |
| 7 | RATIO Z | 0.020 | P75 Z | 0.020 | MIN Y | 0.019 | P75 Y | 0.018 | P25 R | 0.018 |
| 8 | MED Z | 0.025 | MED R | 0.025 | RMS R | 0.024 | MEAN R | 0.024 | MAX R | 0.023 |
| 9 | RATIO Z | 0.026 | MED R | 0.024 | RATIO X | 0.024 | MEAN Y | 0.023 | MED Z | 0.023 |
| 10 | P75 X | 0.020 | MED R | 0.018 | MAX Y | 0.018 | P75 Z | 0.018 | SE R | 0.018 |
| 11 | SD Z | 0.020 | P75 R | 0.019 | MAX Y | 0.019 | RATIO Z | 0.017 | SD X | 0.016 |
| 12 | RMS R | 0.025 | MAX R | 0.023 | MIN Z | 0.023 | MED Z | 0.023 | RATIO X | 0.022 |
| 13 | SD Z | 0.017 | P25 R | 0.016 | P75 Z | 0.015 | SE R | 0.014 | P75 Y | 0.014 |
| 14 | MAX R | 0.024 | P75 X | 0.024 | P75 R | 0.023 | MIN Z | 0.023 | P75 Y | 0.023 |
| 15 | P25 Y | 0.014 | SD R | 0.014 | RMS Z | 0.010 | P75 Y | 0.009 | MED R | 0.009 |
| 16 | MAX R | 0.023 | SE R | 0.021 | MEAN Z | 0.021 | MAX Y | 0.019 | P25 R | 0.018 |

| Left-out Participant | Variable 26 | Variable 26 Imp. | Variable 27 | Variable 27 Imp. | Variable 28 | Variable 28 Imp. | Variable 29 | Variable 29 Imp. | Variable 30 | Variable 30 Imp. |
| --- | --- | --- | --- | --- | --- | --- | --- | --- | --- | --- |
| 1 | MAX R | 0.018 | P25 Y | 0.017 | P25 Z | 0.017 | MEAN Y | 0.015 | RMS X | 0.015 |
| 2 | MIN Y | 0.020 | P75 Z | 0.020 | P75 X | 0.019 | P75 Y | 0.018 | MIN X | 0.018 |
| 3 | P25 R | 0.019 | MEAN X | 0.018 | P75 X | 0.018 | MIN Y | 0.017 | P25 Z | 0.015 |
| 4 | P25 Z | 0.020 | SD X | 0.020 | MEAN Z | 0.018 | MEAN Y | 0.018 | RMS X | 0.017 |
| 5 | P75 Y | 0.015 | P25 Y | 0.015 | SE R | 0.14 | P25 Z | 0.012 | SD X | 0.012 |
| 6 | MIN Y | 0.018 | MAX R | 0.017 | P25 Y | 0.016 | MAX Z | 0.016 | SD X | 0.015 |
| 7 | SE R | 0.018 | MEAN Z | 0.017 | P25 Y | 0.016 | P25 Z | 0.015 | MIN X | 0.015 |
| 8 | P25 R | 0.022 | MEAN Y | 0.022 | RMS X | 0.021 | P75 Y | 0.021 | P25 Y | 0.020 |
| 9 | MIN Z | 0.022 | MAX R | 0.022 | MAX Y | 0.022 | P25 Y | 0.021 | P75 Y | 0.020 |
| 10 | SD R | 0.017 | P25 Y | 0.015 | RMS X | 0.014 | P25 Z | 0.014 | SD X | 0.014 |
| 11 | SE R | 0.015 | MEAN Z | 0.015 | P75 X | 0.014 | MIN X | 0.014 | P25 Z | 0.012 |
| 12 | MED R | 0.021 | MEAN Y | 0.021 | MEAN R | 0.020 | MEAN X | 0.019 | P75 Z | 0.018 |
| 13 | MIN X | 0.013 | RATIO Z | 0.013 | MIN Y | 0.012 | P75 X | 0.010 | P25 Z | 0.010 |
| 14 | MED Z | 0.023 | MED R | 0.022 | MEAN Y | 0.022 | SD X | 0.021 | RMS X | 0.021 |
| 15 | MEAN Z | 0.007 | RMS X | 0.006 | SE R | 0.005 | SE Z | 0.005 | P75 X | 0.005 |
| 16 | P75 X | 0.018 | P75 Z | 0.016 | MIN Y | 0.016 | P25 Y | 0.016 | SD X | 0.015 |

| Left-out Participant | Variable 31 | Variable 31 Imp. | Variable 32 | Variable 32 Imp. | Variable 33 | Variable 33 Imp. | Variable 34 | Variable 34 Imp. | Variable 35 | Variable 35 Imp. |
| --- | --- | --- | --- | --- | --- | --- | --- | --- | --- | --- |
| 1 | MIN Y | 0.015 | SD R | 0.014 | P75 Z | 0.014 | MAX Z | 0.012 | MED Z | 0.012 |
| 2 | SD X | 0.016 | P25 Y | 0.015 | RMS X | 0.014 | MEAN X | 0.014 | MED Z | 0.014 |
| 3 | P75 Z | 0.015 | MEAN Y | 0.015 | RMS X | 0.014 | MIN X | 0.014 | P25 Y | 0.013 |
| 4 | P75 X | 0.017 | MEAN X | 0.016 | MAX Z | 0.013 | P75 Y | 0.012 | P75 Z | 0.012 |
| 5 | MIN X | 0.011 | MEAN Z | 0.011 | MAX Z | 0.010 | RMS X | 0.009 | MED Y | 0.008 |
| 6 | P25 Z | 0.015 | P75 Y | 0.013 | RMS X | 0.012 | MIN X | 0.012 | MEAN Y | 0.010 |
| 7 | P75 X | 0.014 | SD X | 0.013 | RMS X | 0.011 | MEAN Y | 0.011 | MAX Z | 0.010 |
| 8 | MAX Y | 0.020 | P75 Z | 0.020 | MIN Y | 0.018 | MIN Z | 0.017 | MED Y | 0.017 |
| 9 | SD X | 0.020 | MEAN X | 0.020 | MIN Y | 0.019 | RMS X | 0.019 | P75 Z | 0.019 |
| 10 | MEAN Z | 0.014 | MIN X | 0.011 | MED Z | 0.010 | MEAN X | 0.010 | MEAN Y | 0.009 |
| 11 | P75 Y | 0.012 | RMS X | 0.011 | P25 Y | 0.009 | MAX Z | 0.008 | MED Y | 0.008 |
| 12 | SD X | 0.018 | RMS X | 0.017 | MAX Z | 0.016 | MED Y | 0.016 | P75 Y | 0.016 |
| 13 | MAX Z | 0.009 | RMS X | 0.009 | SD X | 0.009 | MEAN X | 0.009 | MED Z | 0.008 |
| 14 | MIN Y | 0.020 | P75 Z | 0.020 | MAX Y | 0.019 | MEAN X | 0.019 | P25 Y | 0.019 |
| 15 | RATIO Z | 0.004 | P25 Z | 0.004 | MAX Z | 0.003 | SD X | 0.003 | MEAN X | 0.002 |
| 16 | P25 Z | 0.015 | MEAN Y | 0.014 | MEAN X | 0.013 | RMS X | 0.011 | MIN X | 0.010 |

| Left-out Participant | Variable 36 | Variable 36 Imp. | Variable 37 | Variable 37 Imp. | Variable 38 | Variable 38 Imp. | Variable 39 | Variable 39 Imp. |
| --- | --- | --- | --- | --- | --- | --- | --- | --- |
| 1 | MEAN X | 0.010 | MED Y | 0.009 | MIN X | 0.009 | MIN R | 0.006 |
| 2 | MEAN Y | 0.013 | MED Y | 0.013 | MAX Z | 0.011 | MIN R | 0.011 |
| 3 | MAX Z | 0.012 | MED Y | 0.011 | MED Z | 0.011 | MIN R | 0.011 |
| 4 | MED Z | 0.011 | MED Y | 0.011 | MIN R | 0.009 | MIN X | 0.009 |
| 5 | MEAN Y | 0.007 | MED Z | 0.007 | MEAN X | 0.006 | MIN R | 0.004 |
| 6 | MED Y | 0.010 | MEAN X | 0.009 | MED Z | 0.007 | MIN R | 0.007 |
| 7 | MEAN X | 0.008 | MED Z | 0.008 | MED Y | 0.007 | MIN R | 0.007 |
| 8 | MEAN X | 0.017 | MAX Z | 0.015 | MIN X | 0.014 | MIN R | 0.013 |
| 9 | MAX Z | 0.018 | MED Y | 0.017 | MIN X | 0.016 | MIN R | 0.013 |
| 10 | MAX Z | 0.008 | P75 Y | 0.008 | MIN R | 0.007 | MED Y | 0.006 |
| 11 | MEAN X | 0.007 | MEAN Y | 0.006 | MED Z | 0.006 | MIN R | 0.005 |
| 12 | MAX Y | 0.016 | MIN Y | 0.014 | MIN X | 0.012 | MIN R | 0.011 |
| 13 | MED Y | 0.008 | P25 Y | 0.007 | MEAN Y | 0.006 | MIN R | 0.004 |
| 14 | MAX Z | 0.017 | MED Y | 0.017 | MIN X | 0.014 | MIN R | 0.009 |
| 15 | MED Y | 0.002 | MEAN Y | 0.001 | MIN R | 0.000 | MED Z | 0.000 |
| 16 | MED Z | 0.010 | MED Y | 0.009 | MAX Z | 0.008 | MIN R | 0.006 |

*Supplementary Table 8. Variable importance rankings and values from the group-based random forest classifiers for Experiment 1. MAX = maximum, MEAN = mean, MED = median, MIN = minimum, P25 = 25^th^ percentile, P75 = 75^th^ percentile, RATIO = ratio of single-axis RMS to RMS of the resultant axis, RMS = root mean square, SE = sample entropy, SD = standard deviation, R = resultant axis, X = vertical axis, Y = medio-lateral axis, Z = anterior-posterior axis*
