# Supplementary material for: Use of subject-specific models to detect fatigue-related changes in running biomechanics: a random forest approach
Source: Front Sports Act Living. 2023 Dec 21;5:1283316. doi: 10.3389/fspor.2023.1283316 (PMC10768007; doi:10.3389/fspor.2023.1283316)
Supplement: Supplementary file 9 [file Table9.docx]

| Participant | Left-out Trial | Variable 1 | Variable 1 Imp. | Variable 2 | Variable 2 Imp. | Variable 3 | Variable 3 Imp. | Variable 4 | Variable 4 Imp. | Variable 5 | Variable 5 Imp. |
| --- | --- | --- | --- | --- | --- | --- | --- | --- | --- | --- | --- |
| 17 | 1 | STB | 0.253 | ST | 0.234 | CD | 0.229 | SL | 0.150 | VO | 0.133 |
|  | 2 | ST | 0.237 | STB | 0.220 | CD | 0.205 | SL | 0.176 | VO | 0.161 |
|  | 3 | STB | 0.269 | ST | 0.234 | VO | 0.192 | CD | 0.179 | SL | 0.126 |
|  | 4 | STB | 0.234 | VO | 0.219 | ST | 0.208 | CD | 0.188 | SL | 0.151 |
|  | 5 | STB | 0.391 | ST | 0.184 | VO | 0.164 | CD | 0.142 | SL | 0.119 |
| 18 | 1 | ST | 0.482 | CD | 0.181 | STB | 0.179 | VO | 0.137 | SL | 0.021 |
|  | 2 | ST | 0.368 | STB | 0.241 | CD | 0.231 | VO | 0.130 | SL | 0.030 |
|  | 3 | ST | 0.273 | STB | 0.262 | VO | 0.204 | CD | 0.192 | SL | 0.070 |
|  | 4 | ST | 0.439 | STB | 0.224 | CD | 0.167 | VO | 0.158 | SL | 0.013 |
|  | 5 | VO | 0.530 | ST | 0.360 | CD | 0.063 | STB | 0.044 | SL | 0.003 |
| 19 | 1 | STB | 0.334 | VO | 0.191 | ST | 0.176 | SL | 0.150 | CD | 0.149 |
|  | 2 | STB | 0.878 | CD | 0.084 | VO | 0.018 | SL | 0.015 | ST | 0.005 |
|  | 3 | STB | 0.329 | VO | 0.244 | ST | 0.173 | CD | 0.162 | SL | 0.093 |
|  | 4 | STB | 0.324 | CD | 0.203 | ST | 0.182 | VO | 0.156 | SL | 0.135 |
|  | 5 | VO | 0.561 | CD | 0.365 | STB | 0.051 | ST | 0.018 | SL | 0.004 |
| 20 | 1 | CD | 0.248 | VO | 0.207 | ST | 0.197 | SL | 0.174 | STB | 0.174 |
|  | 2 | CD | 0.223 | VO | 0.206 | ST | 0.195 | SL | 0.191 | STB | 0.185 |
|  | 3 | CD | 0.219 | VO | 0.201 | STB | 0.200 | SL | 0.192 | ST | 0.188 |
|  | 4 | CD | 0.251 | VO | 0.217 | STB | 0.184 | ST | 0.182 | SL | 0.166 |
|  | 5 | VO | 0.251 | CD | 0.208 | ST | 0.190 | STB | 0.179 | SL | 0.172 |
| 21 | 1 | ST | 0.329 | CD | 0.275 | SL | 0.163 | VO | 0.120 | STB | 0.114 |
|  | 2 | CD | 0.246 | ST | 0.202 | VO | 0.196 | STB | 0.191 | SL | 0.166 |
|  | 3 | ST | 0.285 | CD | 0.272 | VO | 0.168 | SL | 0.167 | STB | 0.108 |
|  | 4 | ST | 0.295 | CD | 0.286 | SL | 0.192 | VO | 0.128 | STB | 0.100 |
|  | 5 | ST | 0.291 | CD | 0.268 | STB | 0.175 | SL | 0.147 | VO | 0.119 |
| 22 | 1 | STB | 0.290 | ST | 0.281 | CD | 0.227 | VO | 0.131 | SL | 0.072 |
|  | 2 | ST | 0.478 | STB | 0.362 | CD | 0.087 | VO | 0.059 | SL | 0.014 |
|  | 3 | ST | 0.442 | STB | 0.341 | CD | 0.122 | VO | 0.077 | SL | 0.018 |
|  | 4 | ST | 0.276 | STB | 0.254 | CD | 0.203 | VO | 0.191 | SL | 0.076 |
|  | 5 | ST | 0.372 | STB | 0.266 | CD | 0.244 | VO | 0.084 | SL | 0.033 |
| 23 | 1 | CD | 0.273 | SL | 0.262 | VO | 0.191 | STB | 0.175 | ST | 0.100 |
|  | 2 | CD | 0.265 | SL | 0.254 | STB | 0.212 | VO | 0.182 | ST | 0.087 |
|  | 3 | CD | 0.532 | VO | 0.293 | STB | 0.107 | SL | 0.043 | ST | 0.025 |
|  | 4 | CD | 0.283 | SL | 0.216 | STB | 0.191 | VO | 0.179 | ST | 0.131 |
|  | 5 | CD | 0.267 | SL | 0.240 | STB | 0.231 | VO | 0.159 | ST | 0.104 |
| 24 | 1 | ST | 0.310 | STB | 0.208 | CD | 0.205 | SL | 0.170 | VO | 0.108 |
|  | 2 | ST | 0.273 | STB | 0.205 | CD | 0.194 | VO | 0.169 | SL | 0.160 |
|  | 3 | CD | 0.255 | STB | 0.230 | VO | 0.198 | ST | 0.177 | SL | 0.140 |
|  | 4 | CD | 0.280 | ST | 0.255 | STB | 0.176 | SL | 0.160 | VO | 0.129 |
|  | 5 | ST | 0.273 | CD | 0.263 | SL | 0.204 | VO | 0.132 | STB | 0.128 |
| 25 | 1 | ST | 0.319 | CD | 0.259 | VO | 0.199 | SL | 0.123 | STB | 0.100 |
|  | 2 | ST | 0.318 | CD | 0.271 | VO | 0.176 | SL | 0.131 | STB | 0.104 |
|  | 3 | ST | 0.310 | CD | 0.277 | VO | 0.192 | SL | 0.111 | STB | 0.110 |
|  | 4 | ST | 0.290 | CD | 0.240 | VO | 0.215 | SL | 0.139 | STB | 0.117 |
|  | 5 | ST | 0.381 | CD | 0.255 | VO | 0.242 | STB | 0.070 | SL | 0.052 |

*Supplementary Table 9. Variable importance rankings and values from the subject-specific random forest classifiers for Experiment 2. CD = cadence, SL = stride length, ST = stance time, STB = stance time balance, VO = vertical oscillation.*
