# Supplementary material for: Use of subject-specific models to detect fatigue-related changes in running biomechanics: a random forest approach
Source: Front Sports Act Living. 2023 Dec 21;5:1283316. doi: 10.3389/fspor.2023.1283316 (PMC10768007; doi:10.3389/fspor.2023.1283316)
Supplement: Supplementary file 10 [file Table10.docx]

| Left-out Participant | Variable 1 | Variable 1 Imp. | Variable 2 | Variable 2 Imp. | Variable 3 | Variable 3 Imp. | Variable 4 | Variable 4 Imp. | Variable 5 | Variable 5 Imp. |
| --- | --- | --- | --- | --- | --- | --- | --- | --- | --- | --- |
| 17 | CD | 0.578 | ST | 0.329 | STB | 0.044 | SL | 0.025 | VO | 0.023 |
| 18 | CD | 0.337 | ST | 0.208 | SL | 0.164 | VO | 0.160 | STB | 0.131 |
| 19 | CD | 0.330 | ST | 0.262 | SL | 0.186 | STB | 0.125 | VO | 0.097 |
| 20 | ST | 0.620 | CD | 0.309 | SL | 0.049 | VO | 0.019 | STB | 0.004 |
| 21 | CD | 0.491 | ST | 0.332 | STB | 0.101 | SL | 0.039 | VO | 0.038 |
| 22 | CD | 0.323 | ST | 0.246 | STB | 0.169 | SL | 0.155 | VO | 0.107 |
| 23 | ST | 0.508 | CD | 0.390 | STB | 0.044 | SL | 0.044 | VO | 0.014 |
| 24 | CD | 0.308 | ST | 0.261 | SL | 0.175 | STB | 0.149 | VO | 0.107 |
| 25 | CD | 0.344 | ST | 0.287 | STB | 0.148 | SL | 0.143 | VO | 0.078 |

*Supplementary Table 10. Variable importance rankings and values from the group-based random forest classifiers for Experiment 2. CD = cadence, SL = stride length, ST = stance time, STB = stance time balance, VO = vertical oscillation.*
